# Durability of mRNA-1273 vaccine–induced antibodies against SARS-CoV-2 variants

**DOI:** 10.1126/science.abj4176

**Published:** 2021-08-13

**Authors:** Amarendra Pegu, Sarah O’Connell, Stephen D. Schmidt, Sijy O’Dell, Chloe A. Talana, Lilin Lai, Jim Albert, Evan Anderson, Hamilton Bennett, Kizzmekia S. Corbett, Britta Flach, Lisa Jackson, Brett Leav, Julie E. Ledgerwood, Catherine J. Luke, Mat Makowski, Martha C. Nason, Paul C. Roberts, Mario Roederer, Paulina A. Rebolledo, Christina A. Rostad, Nadine G. Rouphael, Wei Shi, Lingshu Wang, Alicia T. Widge, Eun Sung Yang, The mRNA-1273 Study Group, John H. Beigel, Barney S. Graham, John R. Mascola, Mehul S. Suthar, Adrian B. McDermott, Nicole A. Doria-Rose

**Affiliations:** 1 Vaccine Research Center, National Institute of Allergy and Infectious Diseases, National Institutes of Health, Bethesda, MD 20892, USA.; 2 Department of Pediatrics, Division of Infectious Disease, Emory Vaccine Center, Yerkes National Primate Research Center, Emory University School of Medicine, Atlanta, GA 30322, USA.; 3 Emmes Company, Rockville, MD 20850, USA.; 4 Moderna, Inc., Cambridge, MA 02139, USA.; 5 Kaiser Permanente Washington Health Research Institute, Seattle, WA 98101, USA.; 6 Division of Microbiology and Infectious Diseases, National Institute of Allergy and Infectious Diseases, National Institutes of Health, Bethesda, MD 20892, USA.; 7 Hope Clinic, Department of Medicine, Emory University School of Medicine, Decatur, GA 30030, USA.

## Abstract

SARS-CoV-2 mutations may diminish vaccine-induced protective immune responses, particularly as antibody titers wane over time. Here, we assess the impact of SARS-CoV-2 variants B.1.1.7 (Alpha), B.1.351 (Beta), P.1 (Gamma), B.1.429 (Epsilon), B.1.526 (Iota), and B.1.617.2 (Delta) on binding, neutralizing, and ACE2-competing antibodies elicited by the vaccine mRNA-1273 over seven months. Cross-reactive neutralizing responses were rare after a single dose. At the peak of response to the second vaccine dose, all individuals had responses to all variants. Binding and functional antibodies against variants persisted in most subjects, albeit at low levels, for 6-months after the primary series of the mRNA-1273 vaccine. Across all assays, B.1.351 had the lowest antibody recognition. These data complement ongoing studies to inform the potential need for additional boost vaccinations.

SARS-CoV-2, the virus that causes COVID-19, has infected millions of people worldwide fueling the ongoing global pandemic (1). The combination of RNA virus mutation rates, replication and recombination, in a very large number of individuals is conducive to the emergence of viral variants with improved replication capacity and transmissibility, as well as immunological escape. Of particular interest are the Variants of Concern B.1.1.7 (20I/501Y.V1 or Alpha), B.1.351 (20H/501Y.V2 or Beta), P.1 (Gamma; first identified in Brazil), B.1.429 (Cal20 or Epsilon; first identified in California), and B.1.617.2 (Delta; first identified in India); and Variant of Interest B.1.526 (Iota; first identified in New York). In multiple studies, B.1.351 is the most resistant to neutralization by convalescent or vaccinee sera, with 6–15 fold less neutralization activity for sera from individuals immunized with vaccines based on the virus strain first described in January 2020 (Wuhan-Hu-1, spike also called WA1) (2–9). Most of these prior studies evaluated sera from vaccinated individuals at timepoints soon after the first or second dose, and had limited data on the durability of such responses. Likewise, clinical studies have reported somewhat reduced efficacy and effectiveness against the B.1.1.7, B.1.351, and B.1.617.2 variants (10–12). Although such data provide critical insights into the performance of the vaccines against viral variants, they have not fully addressed the durability of cross-reactive binding and functional antibodies.

Here we investigate the impact of SARS-CoV-2 variants on recognition by sera from individuals who received two 100 mcg doses of the SARS-CoV-2 vaccine mRNA-1273. mRNA-1273 encodes the full-length stabilized spike protein of the WA1 and was administered as a two-dose series 28-days apart. We previously described the binding and neutralization activity against the WA1 SARS-CoV-2 spike longitudinally over 7 months from the first vaccination in volunteers from the Phase 1 trial of the mRNA-1273 vaccine (13–16). In the current study, we demonstrate the utility of employing multiple methodologies to assess SARS-CoV-2 vaccine-elicited humoral immunity to variant viruses over time. We tested sera from a random sample of 8 volunteers in each of three age groups: 18–55, 55–70, and 71+ years of age, all of whom had samples available from four timepoints: 4 weeks after the first dose, and two weeks, 3 months, and 6 months after the second dose (Days 29, 43, 119, and 209 after the first dose, respectively).

Three functional assays and two binding assays were used to assess the humoral immune response to the SARS-CoV-2 spike protein. SARS-CoV-2 neutralization was measured using both a lentivirus-based pseudovirus assay, and a live-virus focus reduction neutralization test (FRNT) (17). The third functional assay was a MSD-ECLIA (Meso Scale Discovery-Electrochemiluminescence immunoassay)-based ACE2 competition assay. This method measured the ability of mRNA-1273 vaccine-elicited antibodies to compete with labeled soluble ACE2 for binding to the specific RBD (WA1 or variant) spotted onto the MSD plate. Antibody binding to cell-surface expressed full-length spike was analyzed by flow cytometry. Binding to soluble protein was measured by interferometry in the MSD-ECLIA platform. All samples were assessed against WA1 and the B.1.1.7 and B.1.351 variants in each of these orthogonal serology assays. In addition, all samples were tested against WA1 containing the D614G mutation in both neutralization assays, as well as binding in the cell-surface assay. Further variants were tested in binding assays as follows: S-2P and RBD binding, P.1 against all samples; cell-surface spike binding, P.1, B.1.429, B.1.526, and B.1.617.2 against all samples. A subset of samples - Day 43 to capture the peak response, and Day 209 to look at durability - were evaluated by pseudovirus neutralization against P.1, B.1.429, B.1.526, and B.1.617.2. The specific sequences used in each assay are defined in table S1.

We first assessed the patterns of antibody activity over time. Consistently across assays, low-level recognition of all variants was observed after a single dose (Day 29) (Fig. 1). Activity against all variants peaked two weeks after the second dose (Day 43) with moderate declines over time through Day 209 (Fig. 1). Notably, the values obtained for each assay on a per-sample basis correlated with each other (fig. S1). We next evaluated the relative impact of each variant, considering all timepoints together. Employing the pseudovirus assay, the neutralizing activity was highest against D614G and lowest against B.1.351, with values for all other variants tested falling in between those two variants (Fig. 1A and Fig. 2A). Similar to previous reports from our group (15) and others (18), pseudovirus neutralization ID50s to D614G were 3-fold higher than to WA1 (Fig. 2G). In contrast, using the live-virus FRNT neutralization assay (Fig. 1B and Fig. 2B), titers to WA1 were higher than to D614G, consistent with previous reports for that assay (19). For all other variants, the impact in the live-virus and pseudovirus neutralization assays were concordant: titers against B.1.1.7 were similar to D614G and lower against B.1.351. ACE2 competition was highest for WA1 RBD, intermediate for B.1.1.7, and lowest for B.1.351 (Fig. 1C and Fig. 2C). Spike-binding antibodies were measured using two different methodologies. In the cell-surface spike binding assay, serum antibodies were bound to full-length, membrane-embedded spike on the surface of transfected cells and measured by flow cytometry (20). In this assay (Fig. 1D and Fig. 2D), WA1 and D614G were nearly indistinguishable, with ~1.5-fold reduced binding to B.1.1.7, B.1.526, B.1.617.2, and 2.4 to 3.0-fold reduced binding to P.1, and B.1.429, and B.1.351. We also used the MSD-ECLIA multiplex binding assay to simultaneously measure IgG binding against both the stabilized soluble spike protein S-2P (21) and RBD proteins derived from WA1 and the B.1.1.7, B.1.351, and P.1 variants. The ECLIA assay showed slightly reduced binding to the variant S-2P (Fig. 1E and Fig. 2E) and RBD (Fig. 1F and Fig. 2F) proteins, with the rank order of highest to lowest binding as follows: WA1, B.1.1.7, P.1, and B.1.351. The overall effect of each variant in each assay is tabulated in Fig. 2G, which shows the geometric mean of the ratios between values for WA1 and variant or D614G and variant. In all assays, B.1.351 was the variant that caused the greatest reduction in titers compared to WA1 or D614G.

To quantify the breadth of responses, we calculated the number of sera that maintained detectable antibody titers in each assay and timepoint (Fig. 3). Antibodies that bound to S-2P and RBD of WA1, B.1.1.7, B.1.351, and P.1 sequences were detected in all subjects at all timepoints. Likewise, binding to full-length cell-surface expressed spike was detected against WA1, D614G, and all six variants at all timepoints. In contrast, the functional assays revealed deficits in antibody recognition of the variants. In the pseudovirus neutralization assay, consistent with our previous report (13), 25% of sera neutralized WA1 after 1 dose (Day 29). 83% of Day 29 sera neutralized D614G, which is more sensitive than WA1 in this assay as noted above; but 33% neutralized B.1.1.7 and only 8% could neutralize B.1.351. Similarly, in the live-virus assay, most Day 29 sera neutralized WA1, D614G and B.1.1.7 but only 8% neutralized B.1.351; and ACE2 competition of binding to B.1.351 RBD was detected in 63% of sera. While a single dose of mRNA-1273 vaccine provides partial protection against COVID-19 disease in the interval prior to the second vaccination (22), and similar data were reported for the mRNA vaccine BNT162b2 (10, 11), this observation of limited neutralizing magnitude and breadth after one dose underscores the importance of the full two-dose regimen of an mRNA vaccine for protection against SARS-CoV-2 variants.

Two weeks after the second dose (Day 43), all sera neutralized all of the pseudoviruses. Responses waned over time: all sera from 6-months post-second dose (Day 209) neutralized D614G and B.1.429 in this assay, but fewer sera neutralized the other variants, with 88%, 96%, 96%, 88%, 85%, and 54% of sera neutralizing WA1, B.1.1.7, B.1.617.2, B.1.526, P.1, and B.1.351 respectively. Similarly, using the live-virus assay, all sera were active against WA1, D614G, B.1.1.7, and B.1.351 at Day 43; and at Day 209, all sera neutralized WA1 and D614G, 88% of sera neutralized B.1.1.7, and 58% neutralized B.1.351. Moreover, the ACE2 competition assay showed reduced activity against B.1.351 at the later timepoints (Fig. 3). Collectively, the functional assays revealed a decreased frequency of sera with detectable activity against B.1.351 and other variants after a single dose or 6 months after the second dose. Importantly, all subjects had broadly cross-reactive functional activity against all variants at the peak of the response. Thus, individuals who demonstrate waning immune responses over time are likely to have memory B cells capable of delivering an anamnestic response to those variants in the event of exposure to virus, or potentially with an additional dose of vaccine.

To understand the contributions of individual mutations to the immune escape noted in the variants of concern, we assayed Day 43 sera against pseudoviruses bearing D614G plus N501Y, present in B.1.1.7, P.1, and B.1.351 variants; Y453F, found in mink cluster 5 variants (23, 24); and N439K, which is resistant to some therapeutic monoclonal antibodies (25). None of these mutations showed a significant impact on neutralization by Day 43 sera (fig. S2). In contrast, E484K, present in B.1.351, P.1, and B.1.526, significantly impacted neutralization sensitivity, with a geometric mean 2.4-fold lower ID50 (fig. S2).

Immune responses to vaccination are often weaker in older adults (26). We previously showed that vaccination with mRNA-1273 elicited antibodies to SARS-CoV-2 WA1 in subjects aged 56 to 70 and 71 and older that are as potent (15) and durable (13) as those elicited in adults aged 18–55, with a slight decrease in potency for the oldest subjects in live-virus neutralization (13). Here we observed a trend to lower titers against SARS-CoV-2 spike variants in the oldest individuals at Day 209, with marginally statistically significant differences in some assays for some variants. Differences were small and there was overlap between groups (figs. S3 to S5). Importantly, many subjects in the oldest group retained neutralizing activity against the variants 6-months after the second vaccine dose (fig. S5).

Convalescent individuals develop B cell lineages that mature over time, increasing their activity against SARS-CoV-2 variants (27). To test the hypothesis that a similar phenomenon would occur in mRNA-1273-vaccinated individuals, for each timepoint we calculated the ratios of activity against each variant to WA1; we then compared the ratios at Day 43 to Day 209. The ratio of WA1 to B.1.351 binding was greater at Day 209 than at Day 43 for both S2P and RBD, implying a more rapid decay of B.1.351-recognizing antibodies (Fig. 4). However, the opposite effect was observed for ACE2-competing antibodies and live-virus neutralization against B.1.351. This can also be seen in Fig. 1, wherein the points for variants are closer to each other at Day 43 than at Day 209 for S-2P and RBD binding, but closer to each other at Day 209 in live-virus neutralization. The same patterns were observed for B.1.1.7. No significant differences over time were noted for pseudovirus neutralization or cell-surface spike binding. These data suggest that while binding antibodies to variants decayed faster than antibodies to WA1, the functional antibodies to variants may have diminished more slowly.

mRNA-1273-elicited antibody activity against SARS-CoV-2 variants persisted six months after the second dose, albeit at reduced levels compared to WA1 and D614G, with more than half of subjects maintaining neutralizing activity against B.1.351 at the latest timepoint tested. High levels of binding antibodies recognizing all tested variants, including B.1.351 and B.1.617.2, were maintained in all subjects over this time period. The results from these diverse methodologies also showed similar dynamics over 7 months after the first vaccination. The effects on antibody potency and breadth of a third dose of mRNA vaccine, encoding either the WA1 spike (mRNA-1273) or the B.1.351 spike (mRNA-1273.351) or co-administration of both, is currently under investigation: early results show strong boosting of responses to both D614G and variants by vaccination with either sequence (28). While additional studies will be needed to address the impact of new variants that will surely arise in areas of intense viral infection, our data are encouraging for the use of this vaccine in the face of viral variation.

## Data and materials availability:

All data are available in the main text or the supplementary materials.

## Supplementary Material

Supplementary Materials

## Figures and Tables

**Fig. 1.**
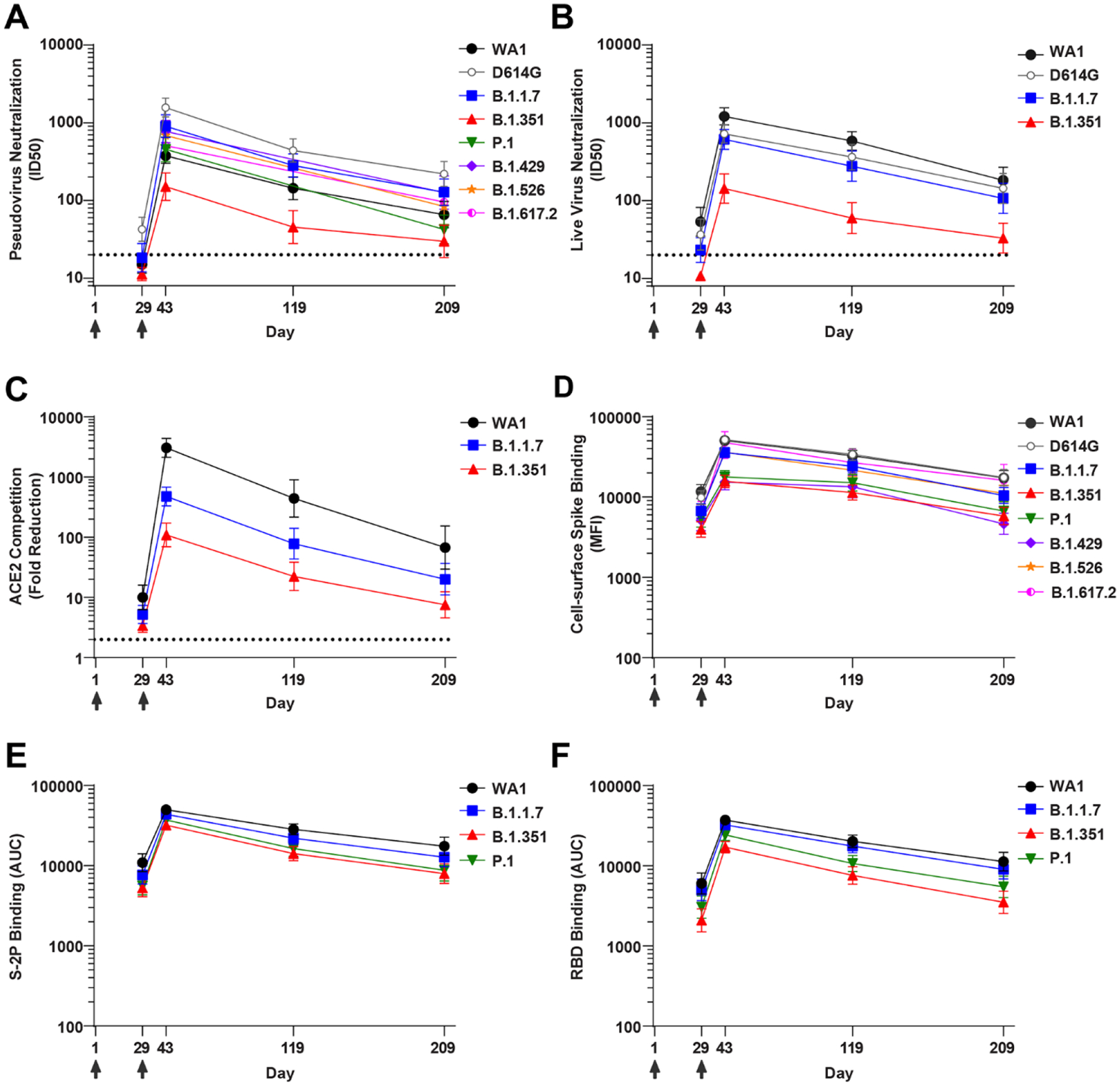
Binding and functional antibodies persist for 6 months following the second dose of the mRNA-1273 vaccine. For all assays, sera from n=24 individuals were sampled at 4 timepoints. Individuals were vaccinated with 100 μg mRNA-1273 at Days 1 and 29 (arrows). Symbols show the geometric mean value; error bars, 95% confidence interval. (**A**) Pseudovirus neutralization, expressed as 50% inhibitory dilution (ID50). Dotted line, limit of detection (>20). Pseudoviruses included WA1, D614G, B.1.1.7, B.1.351, P.1, B.1.429, B.1.526, and B.1.617.2. (**B**) Live-virus FRNT neutralization, expressed as 50% inhibitory dilution (ID50). Dotted line, limit of detection (>20). Viruses included WA1, 83E (spike is D614G), B.1.1.7, and B.1.351. (**C**) Competition of ACE2 binding to RBD, measured by MSD-ECLIA and expressed as fold reduction of ACE2 binding in the presence of serum compared to no-serum control. Dotted line, limit of detection (>2). RBD proteins included WA1, B.1.1.7, and B.1.351. (**D**) Binding to cell-surface expressed full length spike, measured by flow cytometry and expressed as median fluorescence intensity (MFI). Spikes included WA1, D614G, B.1.1.7, B.1.351, P.1, B.1.429, B.1.526, and B.1.617.2. (**E**) Binding to soluble spike protein S-2P, measured by MSD-ECLIA and expressed as area under the curve (AUC). S-2P proteins included WA1, B.1.1.7, B.1.351, and P.1. (**F**) Binding to receptor-binding domain protein (RBD), measured by MSD-ECLIA and expressed as area under the curve (AUC). RBD proteins included WA1, B.1.1.7, B.1.351, and P.1.

**Fig. 2.**
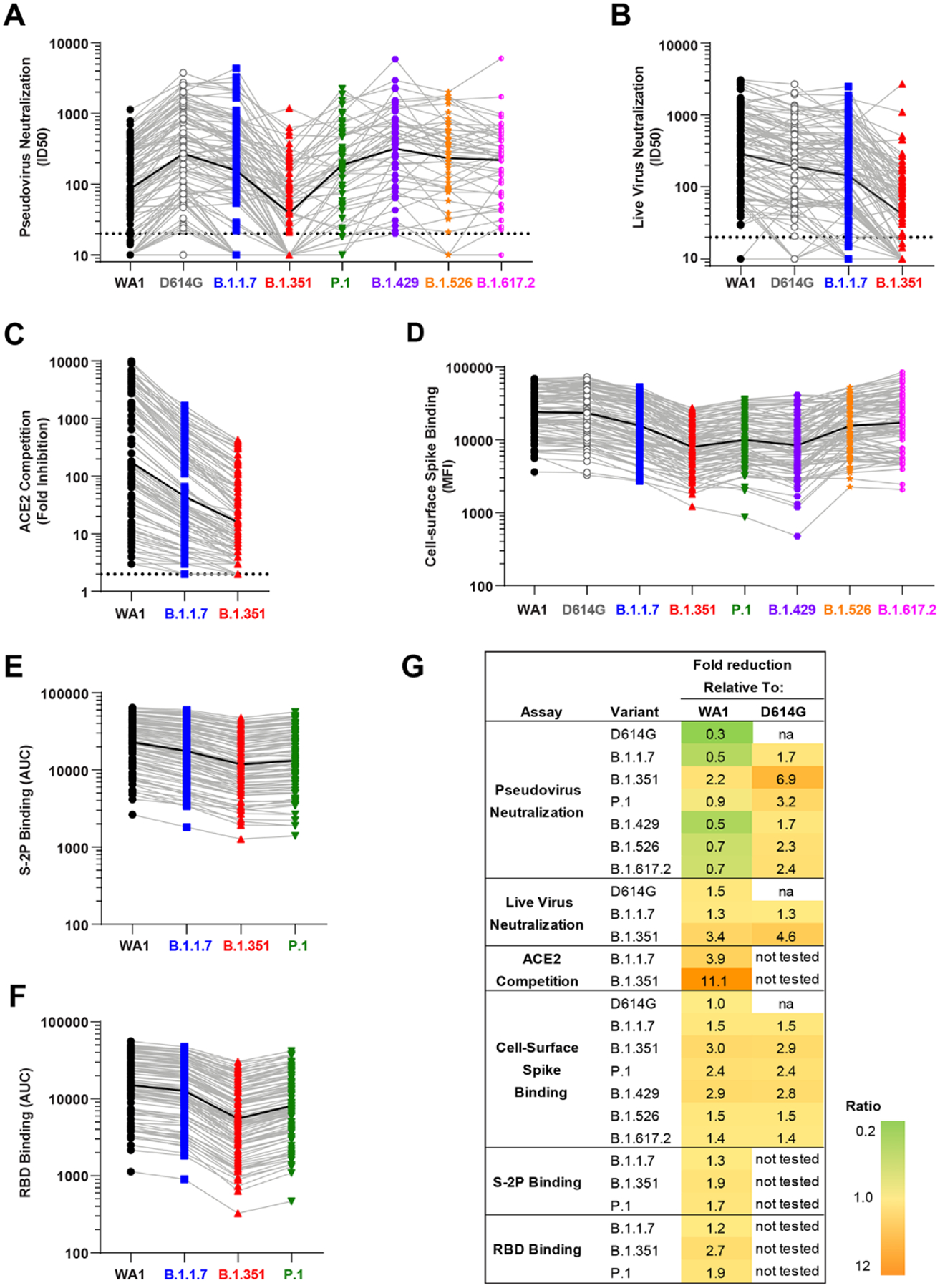
The relative impact of each SARS-CoV-2 viral variant is similar across assays. Symbols show data for all samples at all timepoints; light gray lines connect data from each sample for the variants; black lines show geometric mean of all samples. All viruses are color-coded as in Fig. 1. (**A**) ID50 in pseudovirus neutralization assays. Dotted line, limit of detection (>20). (**B**) ID50 in live virus FRNT neutralization. Dotted line, limit of detection (>20). (**C**) Competition of ACE2 binding to RBD of WA1, B.1.1.7, and B.1.351, expressed as fold reduction of signal in the presence of serum compared to no-serum control. Dotted line, limit of detection (>2). (**D**) Binding to cell-surface expressed full-length spike, expressed as median fluorescence intensity (MFI). (**E**) Binding to S-2P, expressed as area under the curve (AUC). (**F**) Binding to, expressed as AUC. (**G**) Geometric mean of ratios of values. na, not applicable.

**Fig. 3.**
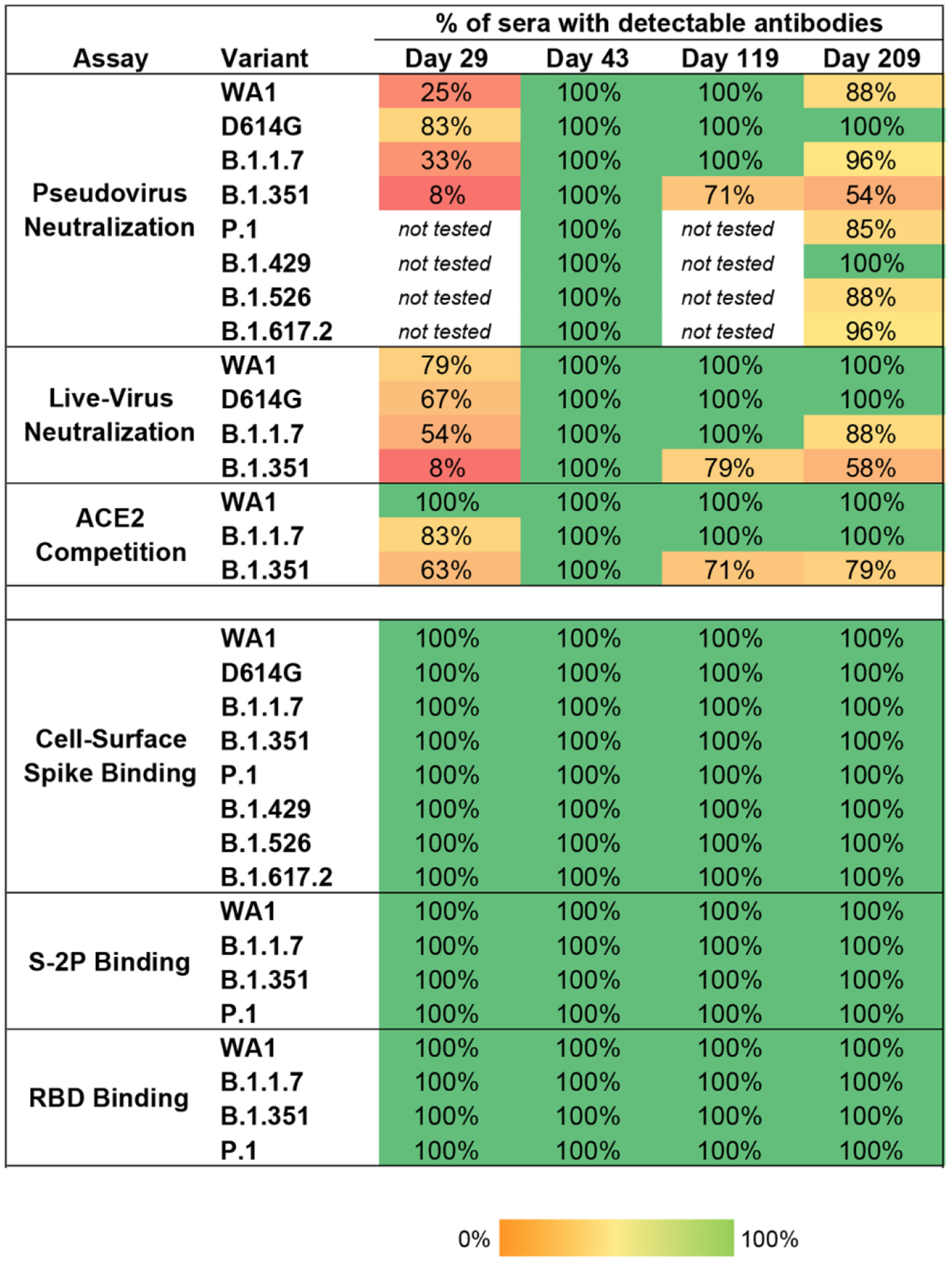
All individuals had binding antibodies to SARS-CoV-2 variants, and the majority of individuals maintained functional activity against viral variants at six months after the second vaccination. Values are the percentage of sera (n=24 at each timepoint) for which antibodies were detected, for each variant. For pseudovirus and live-virus neutralization, samples were called detectable at ID50>20; for ACE2 blocking, at a 2-fold decrease in signal compared to no-serum control; for S-2P and RBD binding, AUC>100; for cell-surface spike binding, MFI>100.

**Fig. 4.**
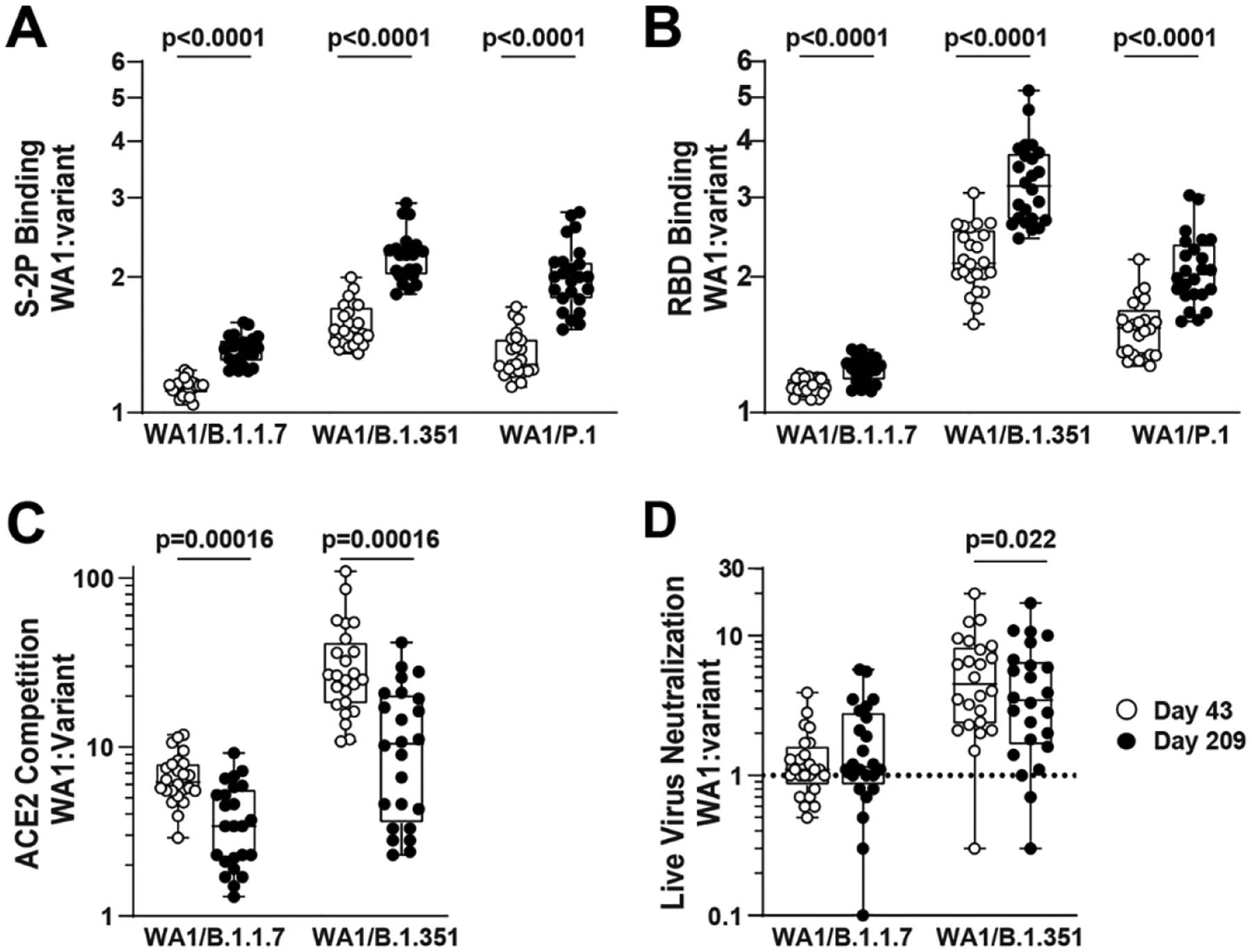
Binding antibodies to viral variants decayed faster than antibodies to WA1, but the functional antibodies to variants diminished more slowly. Symbols show the value for WA1 divided by the value for the indicated variant, for each sample. p values: paired *t* tests. (**A**) S-2P binding. (**B**) RBD binding. (**C**) ACE2 competition. (**D**) Live-virus neutralization.
