# Durability of mRNA-1273 vaccine–induced antibodies against SARS-CoV-2 variants

**DOI:** 10.1126/science.abj4176

**Published:** 2021-09-17

**Authors:** Amarendra Pegu, Sarah E. O’Connell, Stephen D. Schmidt, Sijy O’Dell, Chloe A. Talana, Lilin Lai, Jim Albert, Evan Anderson, Hamilton Bennett, Kizzmekia S. Corbett, Britta Flach, Lisa Jackson, Brett Leav, Julie E. Ledgerwood, Catherine J. Luke, Mat Makowski, Martha C. Nason, Paul C. Roberts, Mario Roederer, Paulina A. Rebolledo, Christina A. Rostad, Nadine G. Rouphael, Wei Shi, Lingshu Wang, Alicia T. Widge, Eun Sung Yang, The mRNA-1273 Study Group, John H. Beigel, Barney S. Graham, John R. Mascola, Mehul S. Suthar, Adrian B. McDermott, Nicole A. Doria-Rose, Jae Arega, John H. Beigel, Wendy Buchanan, Mohammed Elsafy, Binh Hoang, Rebecca Lampley, Aparna Kolhekar, Hyung Koo, Catherine Luke, Mamodikoe Makhene, Seema Nayak, Rhonda Pikaart-Tautges, Paul C. Roberts, Janie Russell, Elisa Sindall, Jim Albert, Pratap Kunwar, Mat Makowski, Evan J. Anderson, Amer Bechnak, Mary Bower, Andres F. Camacho-Gonzalez, Matthew Collins, Ana Drobeniuc, Venkata Viswanadh Edara, Srilatha Edupuganti, Katharine Floyd, Theda Gibson, Cassie M. Grimsley Ackerley, Brandi Johnson, Satoshi Kamidani, Carol Kao, Colleen Kelley, Lilin Lai, Hollie Macenczak, Michele Paine McCullough, Etza Peters, Varun K. Phadke, Paulina A. Rebolledo, Christina A. Rostad, Nadine Rouphael, Erin Scherer, Amy Sherman, Kathy Stephens, Mehul S. Suthar, Mehgan Teherani, Jessica Traenkner, Juton Winston, Inci Yildirim, Lee Barr, Joyce Benoit, Barbara Carste, Joe Choe, Maya Dunstan, Roxanne Erolin, Jana ffitch, Colin Fields, Lisa A. Jackson, Erika Kiniry, Susan Lasicka, Stella Lee, Matthew Nguyen, Stephanie Pimienta, Janice Suyehira, Michael Witte, Hamilton Bennett, Nedim Emil Altaras, Andrea Carfi, Marjorie Hurley, Brett Leav, Rolando Pajon, Wellington Sun, Tal Zaks, Rhea N. Coler, Sasha E. Larsen, Kathleen M. Neuzil, Lisa C. Lindesmith, David R. Martinez, Jennifer Munt, Michael Mallory, Caitlin Edwards, Ralph S. Baric, Nina M. Berkowitz, Eli A. Boritz, Kevin Carlton, Kizzmekia S. Corbett, Pamela Costner, Adrian Creanga, Nicole A. Doria-Rose, Daniel C. Douek, Britta Flach, Martin Gaudinski, Ingelise Gordon, Barney S. Graham, LaSonji Holman, Julie E. Ledgerwood, Kwanyee Leung, Bob C. Lin, Mark K. Louder, John R. Mascola, Adrian B. McDermott, Kaitlyn M. Morabito, Laura Novik, Sarah O’Connell, Sijy O’Dell, Marcelino Padilla, Amarendra Pegu, Stephen D. Schmidt, Wei Shi, Phillip A. Swanson, Chloe A. Talana, Lingshu Wang, Alicia T. Widge, Eun Sung Yang, Yi Zhang, James D. Chappell, Mark R. Denison, Tia Hughes, Xiaotao Lu, Andrea J. Pruijssers, Laura J. Stevens, Christine M. Posavad, Michael Gale, Vineet Menachery, Pei-Yong Shi

**Affiliations:** 1 Vaccine Research Center, National Institute of Allergy and Infectious Diseases, National Institutes of Health, Bethesda, MD 20892, USA.; 2 Department of Pediatrics, Division of Infectious Disease, Emory Vaccine Center, Yerkes National Primate Research Center, Emory University School of Medicine, Atlanta, GA 30322, USA.; 3 Emmes Company, Rockville, MD 20850, USA.; 4 Moderna, Inc., Cambridge, MA 02139, USA.; 5 Kaiser Permanente Washington Health Research Institute, Seattle, WA 98101, USA.; 6 Division of Microbiology and Infectious Diseases, National Institute of Allergy and Infectious Diseases, National Institutes of Health, Bethesda, MD 20892, USA.; 7 Hope Clinic, Department of Medicine, Emory University School of Medicine, Decatur, GA 30030, USA.

## Abstract

Severe acute respiratory syndrome coronavirus 2 (SARS-CoV-2) mutations may diminish vaccine-induced protective immune responses, particularly as antibody titers wane over time. Here, we assess the effect of SARS-CoV-2 variants B.1.1.7 (Alpha), B.1.351 (Beta), P.1 (Gamma), B.1.429 (Epsilon), B.1.526 (Iota), and B.1.617.2 (Delta) on binding, neutralizing, and angiotensin-converting enzyme 2 (ACE2)–competing antibodies elicited by the messenger RNA (mRNA) vaccine mRNA-1273 over 7 months. Cross-reactive neutralizing responses were rare after a single dose. At the peak of response to the second vaccine dose, all individuals had responses to all variants. Binding and functional antibodies against variants persisted in most subjects, albeit at low levels, for 6 months after the primary series of the mRNA-1273 vaccine. Across all assays, B.1.351 had the lowest antibody recognition. These data complement ongoing studies to inform the potential need for additional boost vaccinations.

## A boost for boosters

The evolution of severe acute respiratory syndrome coronavirus 2 (SARS-CoV-2) variants of concern poses a potential obstacle to achieving vaccine-induced immunity. Pegu *et al*. examined how viral variants, including the B.1.351 (Beta) and B.1.617.2 (Delta) variant, affected the immune response in a small number of individuals who received the Moderna mRNA-1273 vaccine. By analyzing sera obtained 6 months after the second shot in the primary vaccine series, the researchers found that neutralizing antibody titers persisted against all variants tested. However, neutralizing antibodies against the B1.351 variant had dropped considerably by 6 months, and some individuals had weak, and in some cases no, neutralizing activity. These data may help to guide public health policies regarding additional booster vaccinations. —PNK

## Teaser

Most individuals vaccinated with mRNA-1273 develop functional antibodies against SARS-CoV-2 variants for at least 6 months.

Severe acute respiratory syndrome coronavirus 2 (SARS-CoV-2), the virus that causes COVID-19, has infected millions of people worldwide, fueling the ongoing global pandemic (*1*). The combination of RNA virus mutation rates, replication, and recombination in a very large number of individuals is conducive to the emergence of viral variants with improved replication capacities and transmissibilities as well as increased immunological escape. Of particular interest are the variants of concern B.1.1.7 (20I/501Y.V1 or Alpha), B.1.351 (20H/501Y.V2 or Beta), P.1 (Gamma), and B.1.617.2 (Delta) and the variants of interest B.1.526 (Iota) and B.1.429 (formerly called Epsilon). In multiple studies, B.1.351 was the most resistant to neutralization by convalescent or vaccinee sera, with 6- to 15-fold less neutralization activity for sera from individuals immunized with vaccines based on the virus strain first described in January 2020 (Wuhan-Hu-1, spike also called WA1) (*2*–*9*). Most of these prior studies have evaluated sera from vaccinated individuals at time points soon after the first or second dose and have had limited data on the durability of such responses. Likewise, clinical studies have reported somewhat reduced efficacy and effectiveness against the B.1.1.7, B.1.351, and B.1.617.2 variants (*10*–*12*). Although such data provide critical insights into the performance of the vaccines against viral variants, they have not fully addressed the durability of cross-reactive binding and functional antibodies.

Here, we investigate the effect of SARS-CoV-2 variants on recognition by sera from individuals who received two 100-μg doses of the SARS-CoV-2 vaccine mRNA-1273. mRNA-1273 encodes the full-length stabilized spike protein of the WA1 strain and was administered as a two-dose series, 28 days apart. We previously described the binding and neutralization activity against the WA1 SARS-CoV-2 spike longitudinally over the course of 7 months from the first vaccination in volunteers from the phase 1 trial of the mRNA-1273 vaccine (*13*–*16*). In the current study, we demonstrate the utility of using multiple methodologies to assess SARS-CoV-2 vaccine–elicited humoral immunity to variant viruses over time. We tested sera from a random sample of eight volunteers in each of three age groups—18 to 55, 55 to 70, and 71+ years of age—all of whom had samples available from four time points: 4 weeks after the first dose and 2 weeks, 3 months, and 6 months after the second dose (days 29, 43, 119, and 209 after the first dose, respectively).

Three functional assays and two binding assays were used to assess the humoral immune response to the SARS-CoV-2 spike protein. SARS-CoV-2 neutralization was measured using both a lentivirus-based pseudovirus assay and a live-virus focus reduction neutralization test (FRNT) (*17*). The third functional assay was a meso scale discovery–electrochemiluminescence immunoassay (MSD-ECLIA)–based angiotensin-converting enzyme 2 (ACE2) competition assay. This method measured the ability of mRNA-1273 vaccine–elicited antibodies to compete with labeled soluble ACE2 for binding to the specific receptor-binding domain (RBD) (WA1 or variant) spotted onto the MSD plate. Antibody binding to cell surface–expressed full-length spike was analyzed by flow cytometry. Binding to soluble protein was measured by interferometry in the MSD-ECLIA platform. All samples were assessed against WA1 and the B.1.1.7 and B.1.351 variants in each of these orthogonal serology assays. Additionally, all samples were tested against WA1 containing the D614G mutation in both neutralization assays as well as binding in the cell surface assay. Further variants were tested in binding assays as follows: S-2P and RBD binding, P.1 against all samples, and cell surface spike binding, P.1, B.1.429, B.1.526, and B.1.617.2 against all samples. A subset of samples—from day 43 to capture the peak response and day 209 to look at durability—were evaluated by pseudovirus neutralization against P.1, B.1.429, B.1.526, and B.1.617.2. The specific sequences used in each assay are defined in table S1.

**Fig. 1.**
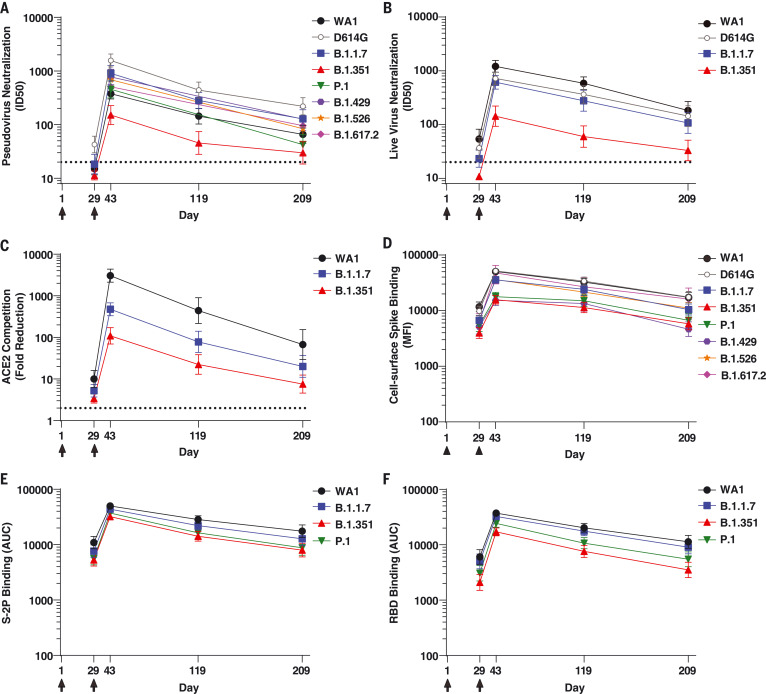
Binding and functional antibodies persist for 6 months after the second dose of the mRNA-1273 vaccine. (**A**) Pseudovirus neutralization, expressed as 50% inhibitory dilution (ID_50_). Dotted line indicates the limit of detection (>20). Pseudoviruses included WA1, D614G, B.1.1.7, B.1.351, P.1, B.1.429, B.1.526, and B.1.617.2. (**B**) Live-virus FRNT neutralization, expressed as ID_50_. Dotted line indicates the limit of detection (>20). Viruses included WA1, 83E (spike is D614G), B.1.1.7, and B.1.351. (**C**) Competition of ACE2 binding to RBD, measured by MSD-ECLIA and expressed as fold reduction of ACE2 binding in the presence of serum compared with no-serum control. Dotted line indicates the limit of detection (>2). RBD proteins included WA1, B.1.1.7, and B.1.351. (**D**) Binding to cell surface–expressed full-length spike, measured by flow cytometry and expressed as median fluorescence intensity (MFI). Spikes included WA1, D614G, B.1.1.7, B.1.351, P.1, B.1.429, B.1.526, and B.1.617.2. (**E**) Binding to soluble spike protein S-2P, measured by MSD-ECLIA and expressed as area under the curve (AUC). S-2P proteins included WA1, B.1.1.7, B.1.351, and P.1. (**F**) Binding to RBD protein, measured by MSD-ECLIA and expressed as AUC. RBD proteins included WA1, B.1.1.7, B.1.351, and P.1. For all assays, sera from *n* = 24 individuals were sampled at four time points. Individuals were vaccinated with 100 μg mRNA-1273 at days 1 and 29 (arrows). Symbols show the geometric mean value, and error bars show 95% confidence intervals.

**Fig. 2.**
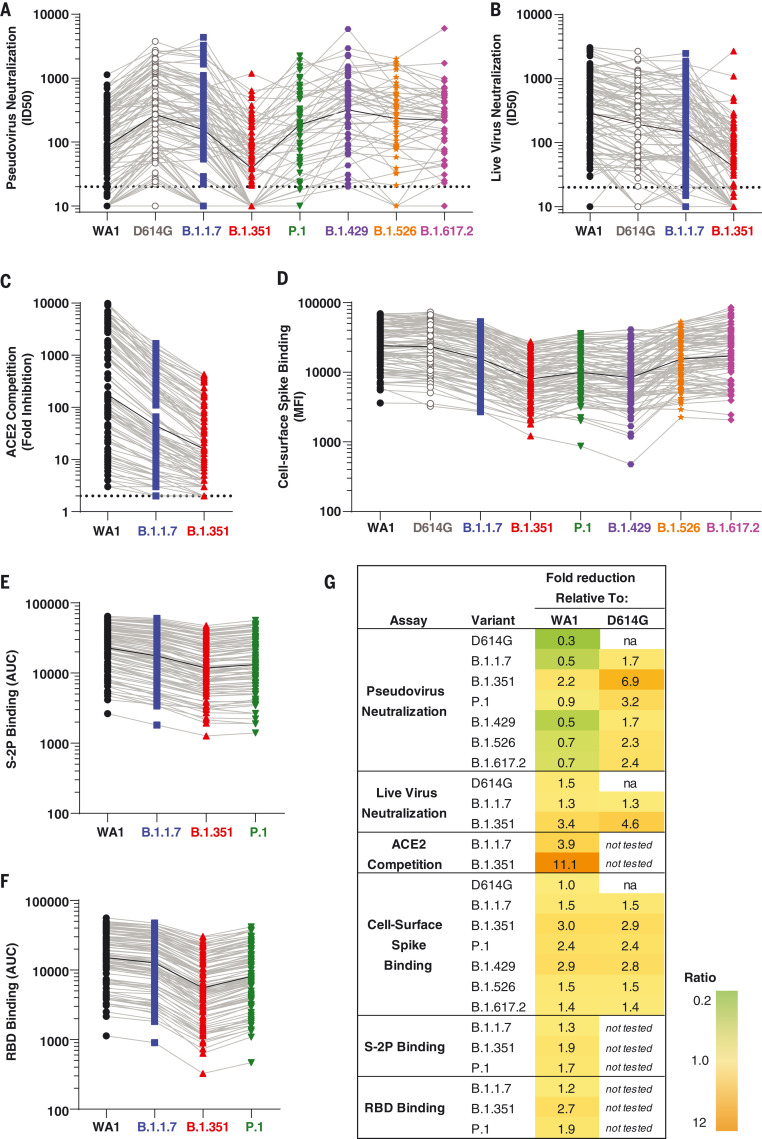
The relative effect of each SARS-CoV-2 viral variant is similar across assays. (**A**) ID_50_ in pseudovirus neutralization assays. Dotted line indicates the limit of detection (>20). (**B**) ID_50_ in live virus FRNT neutralization. Dotted line indicates the limit of detection (>20). (**C**) Competition of ACE2 binding to RBD of WA1, B.1.1.7, and B.1.351, expressed as fold reduction of signal in the presence of serum compared with no-serum control. Dotted line indicates the limit of detection (>2). (**D**) Binding to cell surface–expressed full-length spike, expressed as MFI. (**E**) Binding to S-2P, expressed as AUC. (**F**) Binding to RBD protein, expressed as AUC. (**G**) Geometric mean of ratios of values. na, not applicable. In (A) to (F), symbols show data for all samples at all time points, light gray lines connect data from each sample for the variants, and black lines show geometric mean of all samples. All viruses are color coded as in Fig. 1.

We first assessed the patterns of antibody activity over time. Consistently across assays, low-level recognition of all variants was observed after a single dose (day 29) (Fig. 1). Activity against all variants peaked 2 weeks after the second dose (day 43), with moderate declines over time through day 209 (Fig. 1). Notably, the values obtained for each assay on a per-sample basis correlated with each other (fig. S1). We next evaluated the relative effect of each variant, considering all time points together. Using the pseudovirus assay, the neutralizing activity was highest against D614G and lowest against B.1.351, with values for all other variants tested falling between those two variants (Fig. 1A and Fig. 2A). Similar to previous studies from our group (*15*) and others (*18*), pseudovirus neutralization titers to D614G were threefold higher than to WA1 (Fig. 2G). By contrast, using the live-virus FRNT neutralization assay (Fig. 1B and Fig. 2B), titers to WA1 were higher than those to D614G, consistent with previous studies of that assay (*19*). For all other variants, the effect in the live-virus and pseudovirus neutralization assays were concordant: titers against B.1.1.7 were similar to D614G and lower against B.1.351. ACE2 competition was highest for WA1 RBD, intermediate for B.1.1.7, and lowest for B.1.351 (Fig. 1C and Fig. 2C). Spike-binding antibodies were measured using two different methodologies. In the cell surface spike binding assay, serum antibodies were bound to full-length, membrane-embedded spike on the surface of transfected cells and measured by flow cytometry (*20*). In this assay (Fig. 1D and Fig. 2D), WA1 and D614G were nearly indistinguishable, with ~1.5-fold reduced binding to B.1.1.7, B.1.526, and B.1.617.2 and 2.4- to 3.0-fold reduced binding to P.1, B.1.429, and B.1.351. We also used the MSD-ECLIA multiplex binding assay to simultaneously measure immunoglobulin G (IgG) binding against both the stabilized soluble spike protein S-2P (*21*) and RBD proteins derived from WA1 and the B.1.1.7, B.1.351, and P.1 variants. The ECLIA assay showed slightly reduced binding to the variant S-2P (Fig. 1E and Fig. 2E) and RBD (Fig. 1F and Fig. 2F) proteins, with the rank order of highest to lowest binding as follows: WA1, B.1.1.7, P.1, and B.1.351. The overall effect of each variant in each assay is tabulated in Fig. 2G, which shows the geometric mean of the ratios between values for WA1 and variant or D614G and variant. In all assays, B.1.351 was the variant that caused the greatest reduction in titers compared with WA1 or D614G.

**Fig. 3.**
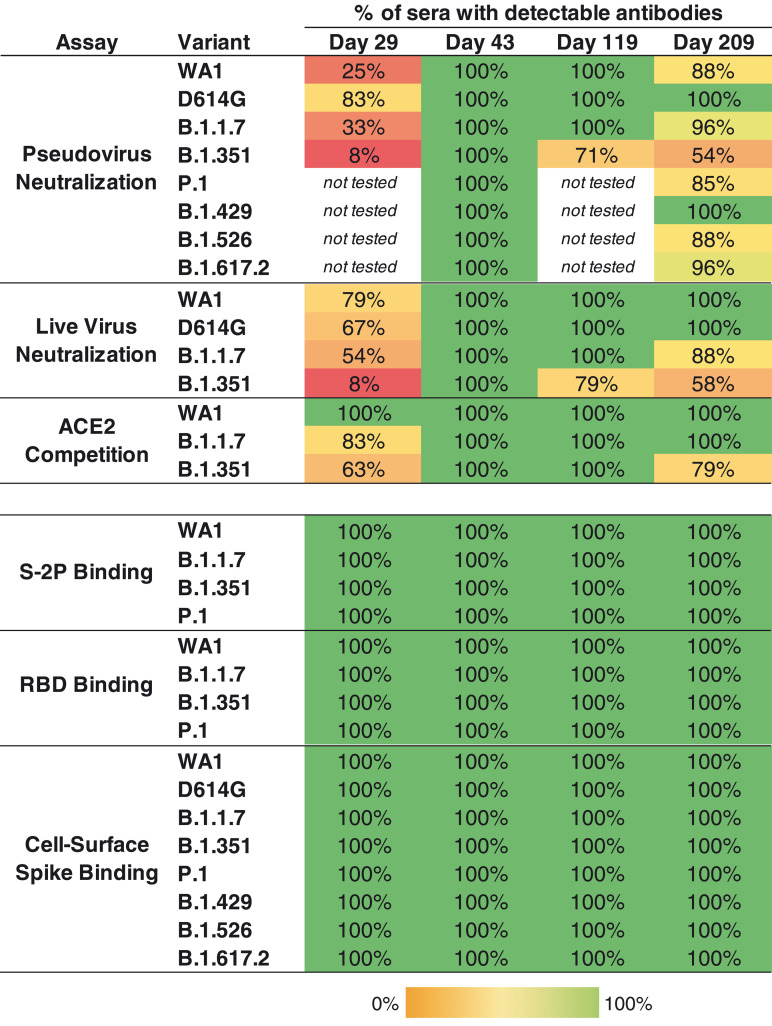
All individuals had binding antibodies to SARS-CoV-2 variants, and the majority of individuals maintained functional activity against viral variants at 6 months after the second vaccination. Values are the percentage of sera (*n* = 24 at each time point) for which antibodies were detected for each variant. For pseudovirus and live-virus neutralization, samples were called detectable at ID_50_ > 20; for ACE2 blocking, at a twofold decrease in signal compared with no-serum control; for S-2P and RBD binding, at AUC > 100; and for cell surface spike binding, at MFI > 100.

To quantify the breadth of responses, we calculated the number of sera that maintained detectable antibody titers in each assay and time point (Fig. 3). Antibodies that bound to S-2P and RBD of the WA1, B.1.1.7, B.1.351, and P.1 sequences were detected in all subjects at all time points. Likewise, binding to full-length cell surface–expressed spike was detected against WA1, D614G, and all six variants at all time points. By contrast, the functional assays revealed deficits in antibody recognition of the variants. In the pseudovirus neutralization assay, consistent with our previous study (*13*), 25% of sera neutralized WA1 after one dose (day 29). In contrast, 83% of day 29 sera neutralized D614G, which is more sensitive than WA1 in this assay as noted above, but 33% neutralized B.1.1.7 and only 8% could neutralize B.1.351. Similarly, in the live-virus assay, most day 29 sera neutralized WA1, D614G, and B.1.1.7, but only 8% neutralized B.1.351, and ACE2 competition of binding to B.1.351 RBD was detected in 63% of sera. Although a single dose of mRNA-1273 vaccine provides partial protection against COVID-19 disease in the interval before the second vaccination (*22*)—and similar data were reported for the mRNA vaccine BNT162b2 (*10*, *11*)—this observation of limited neutralizing magnitude and breadth after one dose underscores the importance of the full two-dose regimen of an mRNA vaccine for protection against SARS-CoV-2 variants.

Two weeks after the second dose (day 43), all sera neutralized all of the pseudoviruses. Responses waned over time: All sera from 6 months after the second dose (day 209) neutralized D614G and B.1.429 in this assay, but fewer sera neutralized the other variants, with 88, 96, 96, 88, 85, and 54% of sera neutralizing WA1, B.1.1.7, B.1.617.2, B.1.526, P.1, and B.1.351, respectively. Similarly, using the live-virus assay, all sera were active against WA1, D614G, B.1.1.7, and B.1.351 at day 43, and at day 209, all sera neutralized WA1 and D614G, 88% of sera neutralized B.1.1.7, and 58% neutralized B.1.351. Moreover, the ACE2 competition assay showed reduced activity against B.1.351 at the later time points (Fig. 3). Collectively, the functional assays revealed a decreased frequency of sera with detectable activity against B.1.351 and other variants after a single dose or 6 months after the second dose. Notably, all subjects had broadly cross-reactive functional activity against all variants at the peak of the response. Thus, individuals who demonstrate waning immune responses over time are likely to have memory B cells capable of delivering an anamnestic response to those variants in the event of exposure to virus, or potentially with an additional dose of vaccine.

To understand the contributions of individual mutations to the immune escape noted in the variants of concern, we assayed day 43 sera against pseudoviruses bearing D614G plus N501Y, present in B.1.1.7, P.1, and B.1.351 variants; Y453F, found in mink cluster five variants (*23*, *24*); and N439K, which is resistant to some therapeutic monoclonal antibodies (*25*). None of these mutations showed a significant impact on neutralization by day 43 sera (fig. S2). By contrast, E484K, present in B.1.351, P.1, and B.1.526, significantly affected neutralization sensitivity, with a geometric mean 2.4-fold lower 50% inhibitory dilution (ID_50_) (fig. S2).

Immune responses to vaccination are often weaker in older adults (*26*). We previously showed that vaccination with mRNA-1273 elicited antibodies to SARS-CoV-2 WA1 in subjects aged 56 to 70 and 71 and older that are as potent (*15*) and durable (*13*) as those elicited in adults aged 18 to 55, with a slight decrease in potency for the oldest subjects in live-virus neutralization (*13*). Here, we observed a trend to lower titers against SARS-CoV-2 spike variants in the oldest individuals at day 209, with marginally statistically significant differences in some assays for some variants. Differences were small, and there was overlap between groups (figs. S3 to S5). Notably, many subjects in the oldest group retained neutralizing activity against the variants 6 months after the second vaccine dose (fig. S5).

**Fig. 4.**
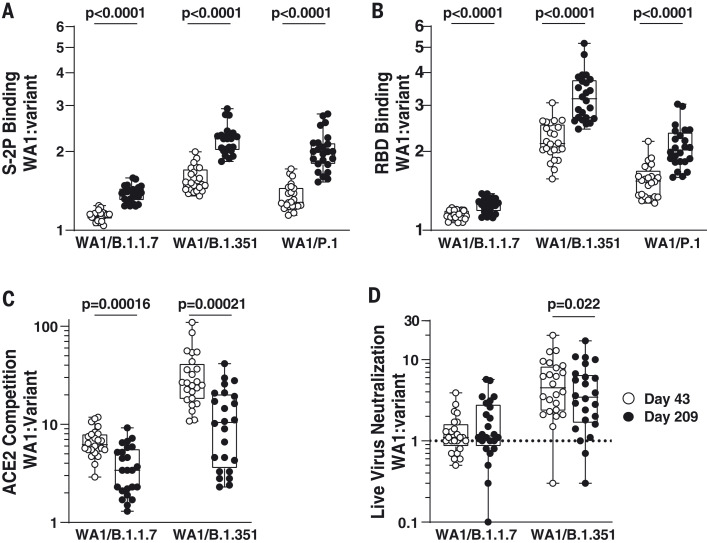
Binding antibodies to viral variants decayed faster than antibodies to WA1, but the functional antibodies to variants diminished more slowly. (**A**) S-2P binding. (**B**) RBD binding. (**C**) ACE2 competition. (**D**) Live-virus neutralization. Symbols show the value for WA1 divided by the value for the indicated variant for each sample. *P* values are from paired *t* tests.

Convalescent individuals develop B cell lineages that mature over time, increasing their activity against SARS-CoV-2 variants (*27*). To test the hypothesis that a similar phenomenon would occur in mRNA-1273–vaccinated individuals, for each time point, we calculated the ratios of activity against each variant to WA1. We then compared the ratios at day 43 with those at day 209. The ratio of WA1 to B.1.351 binding was greater at day 209 than at day 43 for both S2P and RBD, implying a more rapid decay of B.1.351-recognizing antibodies (Fig. 4). However, the opposite effect was observed for ACE2-competing antibodies and live-virus neutralization against B.1.351. This can also be seen in Fig. 1, wherein the points for variants are closer to each other at day 43 than at day 209 for S-2P and RBD binding, but closer to each other at day 209 in live-virus neutralization. The same patterns were observed for B.1.1.7. No statistically significant differences over time were noted for pseudovirus neutralization or cell surface spike binding. These data suggest that although binding antibodies to variants decayed faster than antibodies to WA1, the functional antibodies to variants may have diminished more slowly.

mRNA-1273–elicited antibody activity against SARS-CoV-2 variants persisted 6 months after the second dose, albeit at reduced levels compared with peak activity, with more than half of subjects maintaining neutralizing activity against B.1.351 at the latest time point tested. High levels of binding antibodies recognizing all tested variants, including B.1.351 and B.1.617.2, were maintained in all subjects over this time period. The results from these diverse methodologies also showed similar dynamics over 7 months after the first vaccination. The effects on antibody potency and breadth of a third dose of mRNA vaccine, encoding the WA1 spike (mRNA-1273), the B.1.351 spike (mRNA-1273.351), or coadministration of both, is currently under investigation; early results show strong boosting of responses to both D614G and variants by vaccination with either sequence (*28*). Although additional studies will be needed to address the effect of new variants that will surely arise in areas of intense viral infection, our data are encouraging for the use of this vaccine in the face of viral variation.

## Supplementary Material

20210812-1

## Supplementary Materials

### This PDF file includes:

Materials and Methods Figs. S1 to S6 Table S1 mRNA-1273 Study Group Member List References (*29*–*32*)

### Other Supplementary Material for this manuscript includes the following:

MDAR Reproducibility Checklist

## Notes

View/request a protocol for this paper from *Bio-protocol*.
