# Supplementary material for: Durability of mRNA-1273 vaccine–induced antibodies against SARS-CoV-2 variants
Source: Science. 2021 Aug 12;373(6561):1372–7. doi: 10.1126/science.abj4176 (PMC8691522; doi:10.1126/science.abj4176)
Supplement: Supplementary file 2 — Materials and Methods Figs. S1 to S6 Table S1 mRNA-1273 Study Group Member List References (29–32) [file science.abj4176_sm.pdf]

Science AAAS

Supplementary Materials for

Durability of mRNA-1273 vaccine–induced antibodies against SARS-CoV-2 variants

Amarendra Pegu *et al.*

Corresponding author: Nicole A. Doria-Rose, nicole.doriarose@nih.gov

Science **373**, 1372 (2021)
DOI: 10.1126/science.abj4176

The PDF file includes:

Materials and Methods
Figs. S1 to S6
Table S1
mRNA-1273 Study Group Member List
References

Other Supplementary Material for this manuscript includes the following:

MDAR Reproducibility Checklist

Materials and Methods

Subjects and samples

Subjects in this manuscript participated in a phase 1, dose-escalation, open-label clinical trial of mRNA-1273, as previously reported (13-16). 8 subjects each were randomly chosen from participants from age cohorts 18-55, 56-70, and 71+ years of age who received two doses of 100 mcg mRNA-1273 and for whom samples were available at Days 29, 43, 119, and 209 post first vaccination. The trial was conducted at Kaiser Permanente Washington Health Research Institute in Seattle, WA, the Emory University School of Medicine in Atlanta, GA, and the National Institute of Allergy and Infectious Diseases (NIAID) Vaccine Research Center (VRC) at the National Institutes of Health Clinical Center in Bethesda, MD. Enrolled adults were healthy and provided informed consent prior to any study procedures. Neither PCR nor serology for SARS-CoV-2 was utilized in screening.

Spike sequences

The SARS-CoV-2 Spike sequences used in the assays are shown in Supplemental Table 1.
Live virus 83E differs from WA1 at several positions in the genome, but differs from WA1 in spike only at position 614, hence 83E is used as D614G in these studies.
The exact sequence of B.1.351 spike differed at amino acid 246 between the pseudovirus and live-virus neutralization assays. To address this difference, we compared both spike versions in the pseudovirus assay; overall, there was a 1.3-fold difference, which did not reach statistical significance (Wilcoxon matched-pairs signed rank test) (Figure S6).

Cells and Viruses

VeroE6 cells were obtained from ATCC (clone E6, ATCC, #CRL-1586) and cultured in complete DMEM medium consisting of 1x DMEM (VWR, #45000-304), 10% FBS, 25mM HEPES Buffer (Corning Cellgro), 2mM L-glutamine, 1mM sodium pyruvate, 1x Non-essential Amino Acids, and 1x antibiotics. VeroE6-TMPRSS2 cells were kindly provided by Drs. Barney Graham and Adrian Creanga (Vaccine Research Center, NIH, Bethesda, MD). EHC-083E (D614G SARS-CoV-2) and B.1.1.7 variants were previously described (2, 19). The B.1.351 variant was provided by Dr. Andy Pekosz (John Hopkins University, Baltimore, MD). Viruses were propagated in Vero-TMPRSS2 cells to generate viral stocks. B.1.351 stock was sequenced by Eli Boritz and Daniel Douek (Vaccine Research Center, NIH, Bethesda MD). Viral titers were determined by focus-forming assay on VeroE6 cells. Viral stocks were stored at -80°C until use. Compared to WA1, viral isolate 83E contains the D614G mutation in spike, and several additional mutations elsewhere in the genome.

Pseudovirus neutralization

Neutralization activity against SARS-CoV-2 was measured in a single-round-of-infection assay with pseudotyped virus particles (pseudoviruses) as previously described (15). To produce SARS-CoV-2 pseudoviruses, an expression plasmid bearing codon-optimized SARS-CoV-2 full-length S plasmid was co-transfected into HEK293T/17 cells (ATCC#CRL-11268) cells with packaging plasmid pCMVDR8.2, luciferase reporter plasmid pHR′CMV-Luc (29) and a TMPRSS2 plasmid (30). Spike sequences were: WA1, also called Wuhan-1, Genbank #: MN908947.3; and mutants made in the same plasmid, as in Table 1. Pseudoviruses were mixed with serial dilutions of sera or antibodies and then added to monolayers of ACE2-overexpressing 293T cells (gift of Michael Farzan and Huihui Mu), in triplicate. Three days post infection, cells

were lysed, luciferase was activated with the Luciferase Assay System (Promega), and relative light units (RLU) were measured at 570 nm on a Spectramax L luminometer (Molecular Devices). After subtraction of background RLU (uninfected cells), % neutralization was calculated as 100x((virus only control)-(virus plus antibody))/(virus only control). Dose-response curves were generated with a 5-parameter nonlinear function, and titers reported as the serum dilution or antibody concentration required to achieve 50% (50% inhibitory dilution [ID50]) or 80% (80% inhibitory dilution [ID80]) neutralization. The input dilution of serum is 1:20, thus, 20 is the lower limit of quantification. Samples that do not neutralize at the limit of detection at 50% are plotted at 10, and that value was used for geometric mean calculations. Each assay includes triplicates. In addition, the reported values for WA1 are the geometric mean of 2 independent assays, and D614G values are the geometric mean of 2 or more assays.

Live-virus Neutralization Assay

Live-virus Focus reduction neutralization test (FRNT) assays were performed as previously described (17). Briefly, samples were diluted at 3-fold in 8 serial dilutions using DMEM (VWR, #45000-304) in duplicates with an initial dilution of 1:10 in a total volume of 60 µl. Serially diluted samples were incubated with an equal volume of SARS-CoV-2 (100-200 foci per well) at 37° C for 1 hour in a round-bottomed 96-well culture plate. The antibody-virus mixture was then added to Vero cells and incubated at 37° C for 1 hour. Post-incubation, the antibody-virus mixture was removed and 100 µl of prewarmed 0.85% methylcellulose (Sigma-Aldrich, #M0512-250G) overlay was added to each well. Plates were incubated at 37° C for 24 hours. After 24 hours, methylcellulose overlay was removed, and cells were washed three times with PBS. Cells were then fixed with 2% paraformaldehyde in PBS (Electron Microscopy Sciences) for 30 minutes. Following fixation, plates were washed twice with PBS and 100 µl of permeabilization buffer (0.1% BSA [VWR, #0332], Saponin [Sigma, 47036-250G-F] in PBS), was added to the fixed Vero cells for 20 minutes. Cells were incubated with an anti-SARS-CoV-2 spike primary antibody directly conjugated to biotin (CR3022-biotin) for 1 hour at room temperature. Next, the cells were washed three times in PBS and avidin-HRP was added for 1 hour at room temperature followed by three washes in PBS. Foci were visualized using TrueBlue HRP substrate (KPL, # 5510-0050) and imaged on an ELISPOT reader (CTL). Antibody neutralization was quantified by counting the number of foci for each sample using the Viridot program (31). The neutralization titers were calculated as follows: 1 - (ratio of the mean number of foci in the presence of sera and foci at the highest dilution of respective sera sample). Each specimen was tested in duplicate. The FRNT-50 titers were interpolated using a 4-parameter nonlinear regression in GraphPad Prism 8.4.3. Samples that do not neutralize at the limit of detection at 50% are plotted at 10 and that value was used for geometric mean calculations.

10-plex Meso Scale Discovery-Electrochemiluminescence immunoassay (MSD-ECLIA)

Multiplexed Plates (96 well) precoated with SARS-CoV-2 spike S-2P (WA1), SARS-CoV-2 RBD (WA1), SARS-CoV-2 spike S-2P (B.1.351), SARS-CoV-2 N Protein (WA1), SARS-CoV-2 spike S-2P (B.1.117), SARS-CoV-2 spike S-2P (P.1), SARS-CoV-2 RBD (B.1.351), SARS-CoV-2 RBD (B.1.117), SARS-CoV-2 RBD (P.1) and BSA are supplied by the manufacturer, Meso Scale Diagnostics (Rockville, MD). On the day of the assay, the plate is blocked for 60 minutes with MSD Blocker A (5% BSA). The blocking solution is washed off and test samples

are applied to the wells at 4 dilutions (1:100, 1:500, 1:2500 and 1:10,000) and allowed to incubate with shaking for two hours. Plates are washed and Sulfo-tag labeled anti IgG antibody is applied to the wells and allowed to associate with complexed coated antigen – sample antibody within the assay wells. Plates are washed to remove unbound detection antibody. A read solution containing ECL substrate is applied to the wells, and the plate is entered into the MSD Sector instrument. A current is applied to the plate and areas of well surface where sample antibody has complexed with coated antigen and labeled reporter will emit light in the presence of the ECL substrate. The MSD Sector instrument quantitates the amount of light emitted and reports this ECL unit response as a result for each sample and standard of the plate. Magnitude of ECL response is directly proportional to the extent of binding antibody in the test article. All calculations are performed within Excel and the GraphPad Prism software, version 7.0. Readouts are provided as Area Under Curve (AUC).

ACE2 competition assay

Multiplexed Plates (96 well) precoated with RBD from WA1, B.1.351 and B.1.1.7 SARS-CoV-2 antigen are supplied by the manufacturer. On the day of the assay, the plate is blocked for 30 minutes with MSD Blocker A (5% BSA). The blocking solution is washed off and test samples are applied to the wells at 1:10, 1:20 and 1:40 dilution, in duplicate at each dilution, and allowed to incubate with shaking for one hour. Sulfo-tag labeled ACE2 is applied to the wells and allowed to associate with sample and RBD within the assay wells. Plates are washed to remove unbound detection antibody. A read solution containing ECL substrate is applied to the wells, and the plate is entered into the MSD Sector instrument. A current is applied to the plate and areas of well surface where RBD has complexed with ACE2-SulfoTag will emit light in the presence of the ECL substrate. The MSD Sector instrument quantitates the amount of light emitted and reports this ECL unit response as a result for each sample and standard of the plate. The amount of signal emitted in wells containing no sample (assay diluent only) is evaluated as the maximal binding response. Reduction of ECL response from this maximal readout is directly proportional to the extent of competitive binding activity in the test article. All calculations are performed within Excel and the GraphPad Prism software, version 7.0. Each fold reduction readout is generated against the maximal signal for the matched RBD antigen.

Of all assays used, ACE2 competition showed the greatest relative differences between variants; of note, this methodology depends upon both the relative affinity of the variant RBD sequences for ACE2, and the affinity of the antibodies for binding to RBD at the same site as ACE2. The N501Y mutation, present in all of the tested variant RBD proteins, has been reported to increase affinity of RBD to ACE2 (32). Thus, an antibody with the same binding affinity for WA1 and a variant RBD would be expected to have less capacity to block ACE2 binding to the variant; this will enhance the relative impact of variants on the competition measurement.

Cell-surface spike binding

HEK293T cells were transiently transfected with plasmids encoding full length SARS-CoV-2 spike variants using lipofectamine 3000 (L3000-001, ThermoFisher) following manufacturer's protocol. After 40 hours, the cells were harvested and incubated with serum diluted 1:160 in PBS for 30 minutes. After incubation, the cells were washed and incubated with an allophycocyanin conjugated anti-human IgG (709-136-149, Jackson Immunoresearch Laboratories) along with a

live/dead fixable aqua dead cell stain kit (ThermoFisher) for another 30 minutes. The cells were then washed and fixed with 1% paraformaldehyde (15712-S, Electron Microscopy Sciences). The samples were then acquired on a BD LSR Fortessa X-50 flow cytometer (BD biosciences) and analyzed using Flowjo (BD biosciences). For each serum sample, the median fluorescence intensity (MFI) in the allophycocyanin fluorescence channel was determined for only the spike-transfected cells (typically, 75-90% of all cells).

Statistical analysis

Differences between the assay values between age groups were analyzed using unpaired t tests for log-transformed values, and differences across timepoints were analyzed using paired t tests for log-transformed values. Holm's correction for multiple comparisons was applied to all analyses.

Supplementary Text

mRNA-1273 Study Group

The following study group members were all closely involved with the design, implementation, and oversight of the mRNA-1273 clinical trial.

Division of Microbiology and Infectious Diseases, National Institute of Allergy and Infectious Diseases, National Institutes of Health, Bethesda, MD. Jae Arega, M.S., John H. Beigel, M.D., Wendy Buchanan, M.S., B.S.N., Mohammed Elsafy, M.D., Binh Hoang, Pharm.D., Rebecca Lampley, M.Sc., Aparna Kolhekar, Ph.D., Hyung Koo, B.S.N., Catherine Luke, Ph.D., Mamodikoe Makhene, M.D., M.P.H., Seema Nayak, M.D., Rhonda Pikaart-Tautges, B.S., Paul C. Roberts, Ph.D., Janie Russell, B.S., Elisa Sindall, B.S.N.

The Emmes Company, LLC, Rockville, MD. Jim Albert, M.S., Pratap Kunwar, M.S., Mat Makowski, Ph.D.

Emory University School of Medicine, Atlanta, GA. Evan J. Anderson, M.D., Amer Bechnak, M.D., Mary Bower, R.N., Andres F. Camacho-Gonzalez, M.D., M.Sc., Matthew Collins, M.D., Ph.D., Ana Drobeniuc, M.P.H., Venkata Viswanadh Edara, Ph.D., Srilatha Edupuganti, M.D., M.P.H, Katharine Floyd, Theda Gibson, M.S., Cassie M. Grimsley Ackerley, M.D., Brandi Johnson, Satoshi Kamidani, M.D., Carol Kao, M.D.; Colleen Kelley, M.D., M.P.H., Lilin Lai, M.D., Hollie Macenczak, R.N., Michele Paine McCullough, M.P.H., Etza Peters, R.N., Varun K. Phadke, M.D., Paulina A. Rebolledo, M.D. M.Sc., Christina A. Rostad, M.D., Nadine Rouphael, M.D., Erin Scherer Ph.D., D.Phil., Amy Sherman, M.D., Kathy Stephens, R.N., Mehul S. Suthar, Ph.D., Mehgan Teherani, M.D., M.S., Jessica Traenkner, P.A., Juton Winston, Inci Yildirim, M.D., Ph.D.

Kaiser Permanente Washington Health Research Institute, Seattle, WA. Lee Barr, R.N., Joyce Benoit, R.N., Barbara Carste, M.P.H., Joe Choe, B.S., Maya Dunstan, M.S., R.N., Roxanne

Erolin, M.P.H., Jana ffitch, L.P.N., Colin Fields, M.D., Lisa A. Jackson, M.D., Erika Kiniry, M.P.H., Susan Lasicka, R.Ph., Stella Lee, B.A., Matthew Nguyen, M.P.H., Stephanie Pimienta, B.S., Janice Suyehira, M.D., Michael Witte, Pharm.D.

Moderna, Inc., Cambridge, MA. Hamilton Bennett, M.Sc., Nedim Emil Altaras, Ph.D., Andrea Carfi, Ph.D., Marjorie Hurley, Pharm.D., Brett Leav, M.D., Rolando Pajon, Ph.D., Wellington Sun, M.D., Tal Zaks, M.D., Ph.D.

Seattle Children's Research Institute, Seattle, WA. Rhea N. Coler, M.Sc., Ph.D., Sasha E. Larsen, Ph.D.

University of Maryland School of Medicine, Baltimore, MD. Kathleen M. Neuzil, M.D.

University of North Carolina, Durham, NC. Lisa C. Lindesmith, M.S., David R. Martinez, Ph.D., Jennifer Munt, B.S., Michael Mallory, M.P.H., Caitlin Edwards, B.S., Ralph S. Baric, Ph.D.

Vaccine Research Center, National Institute of Allergy and Infectious Diseases, National Institutes of Health, Bethesda, M.D. Nina M. Berkowitz, M.P.H., Eli Boritz, Ph.D., Kevin Carlton, M.S., Kizzmekia S. Corbett, Ph.D., Pamela Costner, R.N., B.S.N., Adrian Creanga, Ph.D., Nicole A. Doria-Rose, Ph.D., Daniel Douek, Ph.D., Britta Flach, Ph.D., Martin Gaudinski, M.D., Ingelise Gordon, R.N., Barney S. Graham, M.D., LaSonji Holman, F.N.P., Julie E. Ledgerwood, D.O., Kwanyee Leung, Ph.D., Bob C. Lin, B.S., Mark K. Louder, John R. Mascola, M.D., Adrian B. McDermott, Ph.D., Kaitlyn M. Morabito, Ph.D., Laura Novik, R.N., M.A., Sarah O'Connell, M.S., Sijy O'Dell, M.S., Marcelino Padilla, B.S., Amarendra Pegu, Ph.D, Stephen D. Schmidt, B.S., Phillip A. Swanson II, Ph.D., Chloe A. Talana, B.S., Lingshu Wang, Ph.D., Alicia T. Widge, M.D., M.S., Eun Sung Yang M.S., Yi Zhang B.S.

Vanderbilt University Medical Center, Nashville, TN. James D. Chappell, M.D., Ph.D., Mark R. Denison, M.D., Tia Hughes, M.S., Xiaotao Lu, M.S., Andrea J. Pruijssers, Ph.D., Laura J. Stevens, M.S.

Fred Hutchinson Cancer Research Center, Seattle WA. Christine M. Posavad, Ph.D

University of Washington, Seattle, WA. Michael Gale, Jr., Ph.D.

University of Texas Medical Branch, Galveston, TX. Vineet Menachery, Ph.D., Pei-Yong Shi, Ph.D.

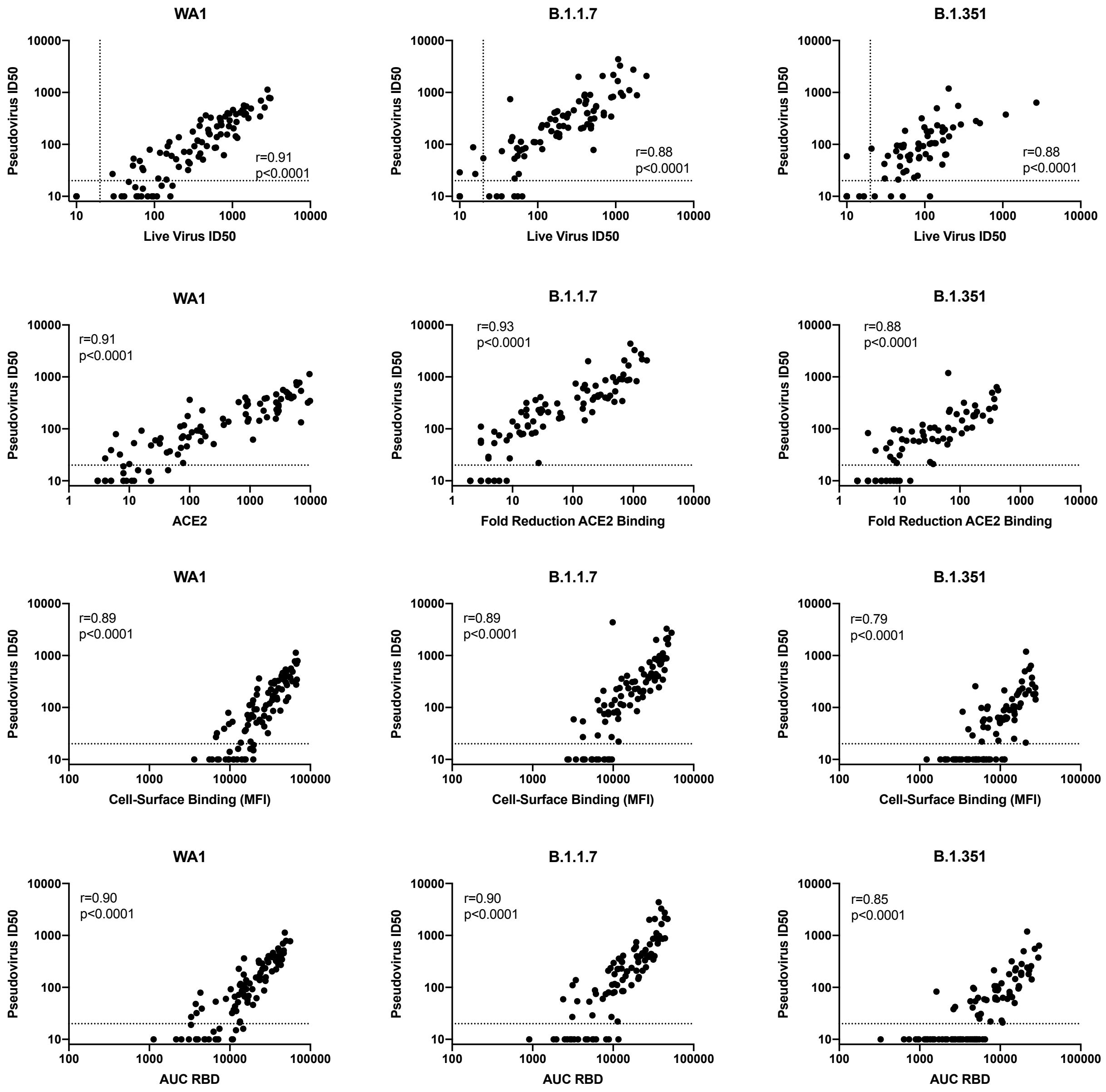

Fig. S1.

Functional and binding assays are highly correlated with each other. Each graph shows n=96 serum samples. r values: Spearman's rho. Graphs show pseudovirus neutralization ID50 of the indicated spike variant compared to: **A.** live-virus FRNT ID50, **B**. fold reduction in ACE2 binding, **C**. cell-surface binding median fluorescence intensity (MFI), **D.** binding to RBD in MSD-ECLIA assay, expressed as area under the curve (AUC).

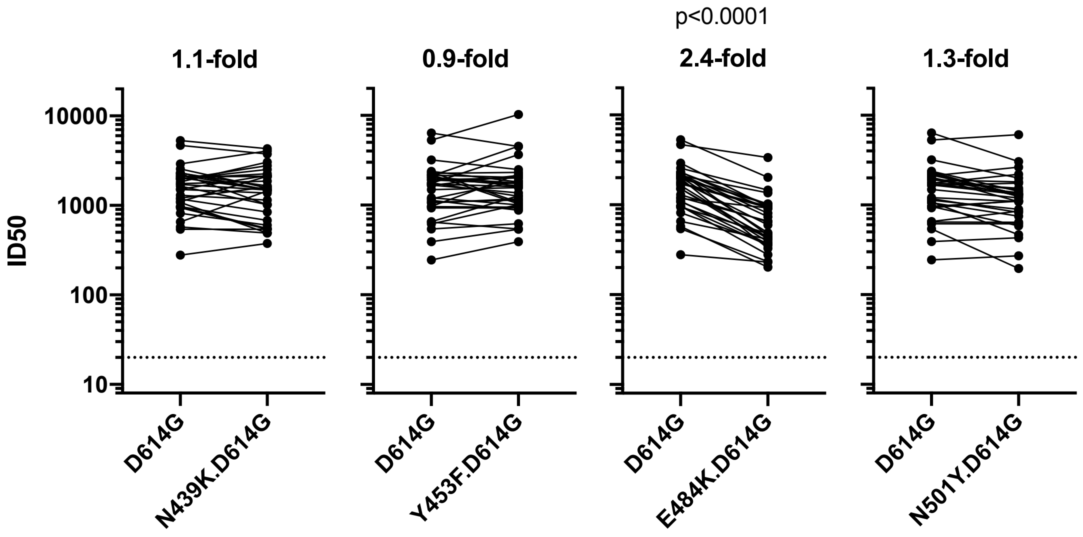

Fig. S2

Point mutations cause modest decreases in neutralizing activity.

Day 43 Sera were assessed in lentivirus-based pseudovirus neutralization assay. 33 sera were tested, inclusive of the 24 used in other figures plus additional samples from subjects who received two doses of 100 µg mRNA1273, as described in (Anderson et al 2020 NEJM). Pseudoviruses were: D614G, D614G.N439K, D614G.Y453F, D614G.E484K, and D614G.N501Y. For each pair of viruses, the fold-difference is the geometric mean of the ratio of ID50s for each serum. P values deteremined by Wilcoxon matched-pairs signed rank test; values <0.05 shown.

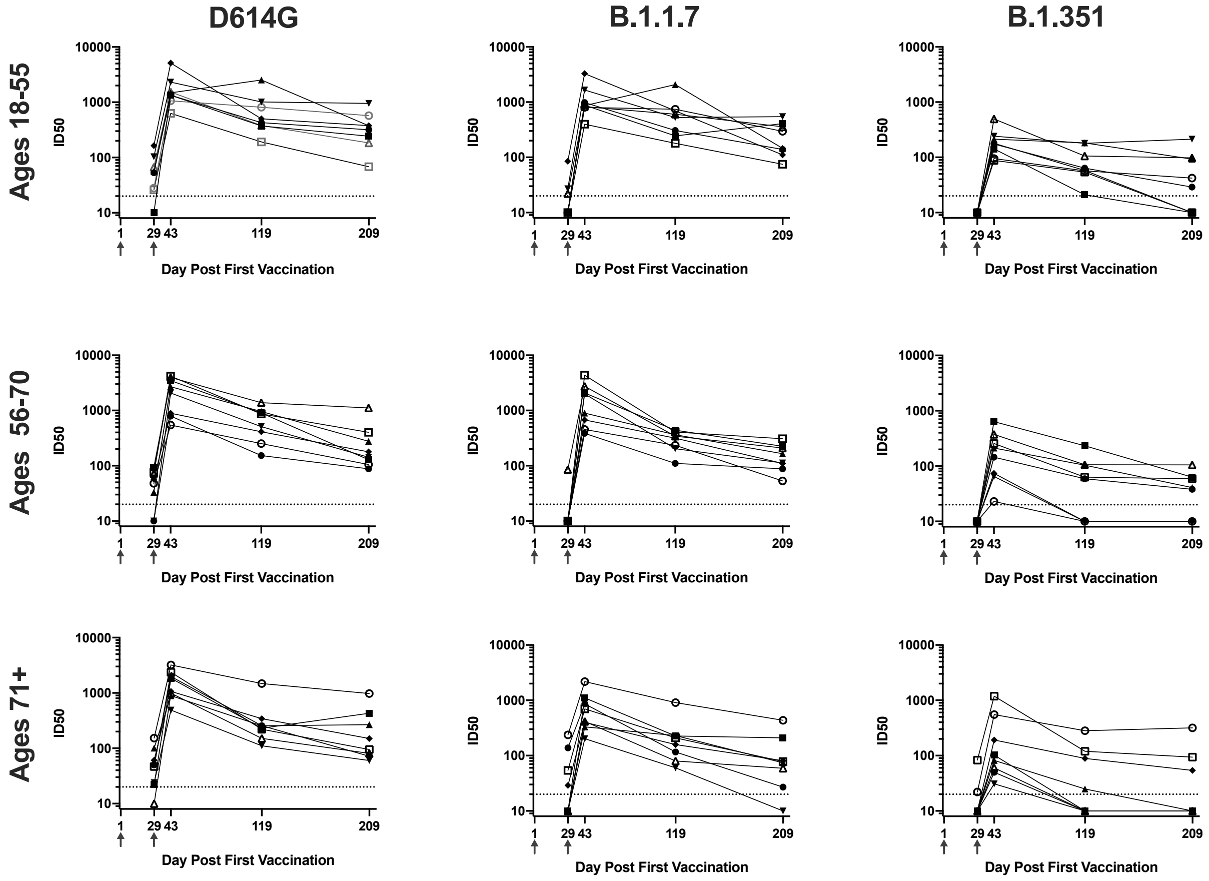

Fig. S3

Neutralization of D614G and variant pseudoviruses is sustained for 6 months.

100 µg mRNA-1273 was delivered at Days 1 and 29 (arrows). Each line represents the pseudovirus neutralization ID50s at Days 29 (4 weeks after first dose), 43 (two weeks after second dose), 119, and 209 for a single subject, n=8 per group. Neutralization activity of sera from subjects aged 18-55 (top row), 56-70 (middle row), and 71+ (bottom row) was measured against pseudoviruses bearing spike of D614G (left), B.1.1.7 (middle), or B.1.351 (right).

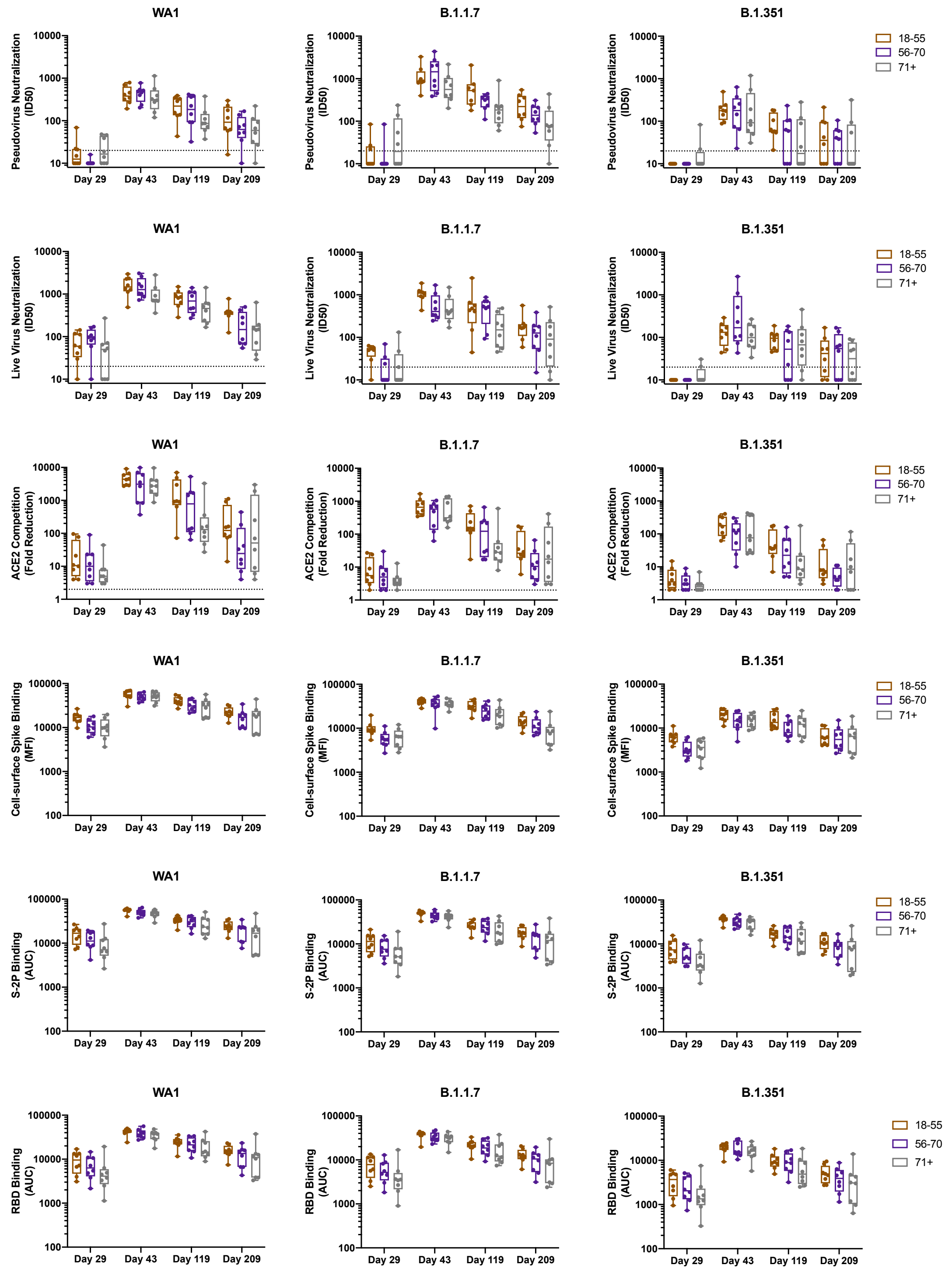

Fig. S4

Effect of age on recognition of variants over time. Assay data are stratified by age group (n=8) and variant. No differences between age groups reached statistical significance for these variants (t-tests of log-transformed values between age groups, with a Holm's correction across the three comparisons).

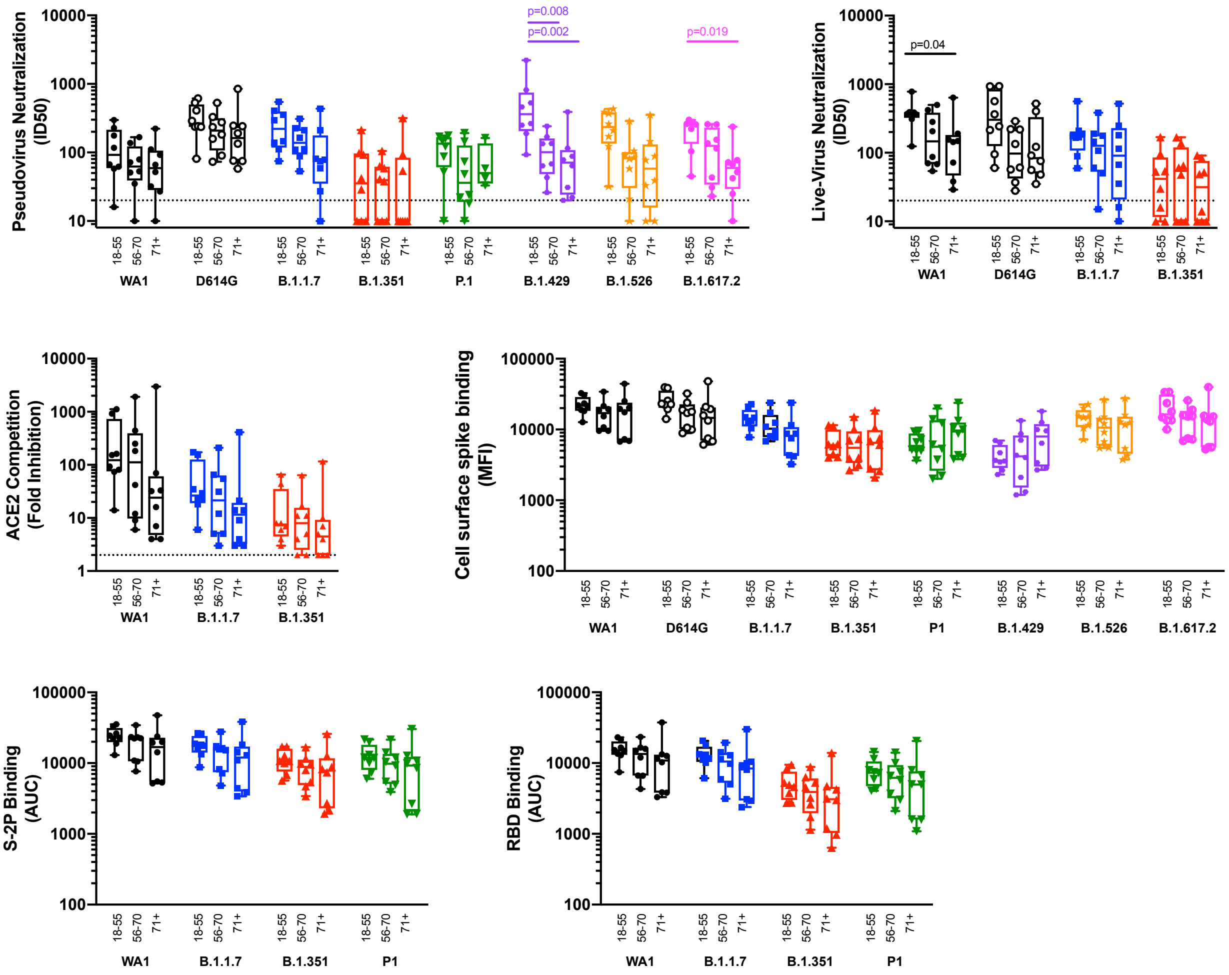

Fig. S5

Effect of age on relative recognition of all variants at Day 209. Assay data are stratified by age group (n=8) and variant. p values: t-tests of log-transformed values between age groups, with a Holm's correction across the three comparisons.

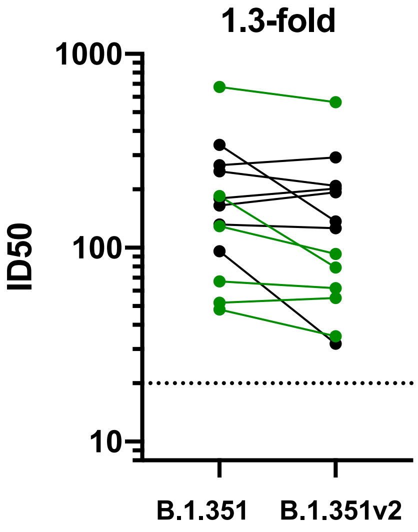

B.1.351	L18F-D80A-D215G-(L242-244)del-R246I-K417N-E484K-N501Y-D614G-A701V
B.1.351v2	L18F-D80A-D215G-(L242-244)del-K417N-E484K-N501Y-D614G-A701V

Fig. S6

Two versions of B.1.351 yield similar pseudovirus neutralization IC50s. N=13 Sera (black, ages 18-55; green, ages 55-70) were assessed in pseudovirus neutralization assay. The spike proteins in the pseudoviruses differ only at amino acid 246 as indicated.

	Name	Mutations relative to parental WA1
**Pseudovirus**	WA1	--
	D614G	D614G
	D614G.N439K	N439K-D614G
	D614G.Y453F	Y453F-D614G
	D614G.E484K	E484K-D614G
	D614G.N501Y	N501Y-D614G
	B.1.1.7	d69H/70V-Y144del-N501Y-A570D-D614G-P681H-T716I-S982A-D1118H
	B.1.351	L18F-D80A-D215G-d(L242-244)-R246I-K417N-E484K-N501Y-D614G-A701V
	B.1.351v2	L18F-D80A-D215G-d(L242-244)-K417N-E484K-N501Y-D614G-A701V
	P.1	L18F-T20N-P26S-D138Y-R190S-K417T-E484K-N501Y-D614G-H655Y-T1027I-V1176F
	B.1.429	S13I-W152C-L425R-D614G
	B.1.526	L5F-T95I-D253G-E484K-D614G-A701V
	B.1.617.2	T19R-G142D-d156/157-R158G-L452R-T478K-D614G-P681R-D950N
**Live Virus**	WA1	--
	83E (D614G)[1]	D614G
	B.1.1.7	d69H/70V-Y144del-N501Y-A570D-D614G-P681H-T716I-S982A-D1118H
	B.1.351[2]	L18F-D80A-D215G-(L242-244)-K417N-E484K-N501Y-D614G-A701V
**Cell-surface expressed Spike**	WA1	--
	D614G	D614G
	B.1.1.7	d69H/70V-dY144-N501Y-A570D-D614G-P681H-T716I-S982A-D1118H
	B.1.351	L18F-D80A-D215G-d(L242-244)-R246I-K417N-E484K-N501Y-D614G-A701V
	P.1	L18F-T20N-P26S-D138Y-R190S-K417T-E484K-N501Y-D614G-H655Y-T1027I-V1176F
	B.1.429	S13I-W152C-L425R-D614G
	B.1.526	L5F-T95I-D253G-E484K-D614G-A701V
	B.1.617.2	T19R-G142D-d156/157-R158G-L452R-T478K-D614G-P681R-D950N
**S-2P Proteins**	WA1	--
	B.1.1.7	d69H/70V-dY144-N501Y-A570D-D614G-P681H-T716I-S982A-D1118H
	B.1.351	L18F-D80A-D215G-d(L242-244)-R246I-K417N-E484K-N501Y-D614G-A701V
	P.1	L18F-T20N-P26S-D138Y-R190S-K417T-E484K-N501Y-D614G-H655Y-T1027I-V1176F
**RBD Proteins**	WA1	--
	B.1.1.7	N501Y
	B.1.351	K417N-E484K-N501Y
	P.1	K417T-E484K-N501Y

d, deletion
[1]Live virus strain 83E differs from WA1 at a single position in Spike, D614G; there are other differences across the genome, as reported in (Edara et al, 2021 JAMA)
[2]Live virus strain B.1.351 has same spike mutations as B.1.351v2 used for pseudoviruses.

Table S1.

Sequences of spike proteins used in each assay.

References and Notes

1. COVID-19 Dashboard by the Center for Systems Science and Engineering (CSSE) at Johns Hopkins University (JHU) (2021); https://coronavirus.jhu.edu/map.html.

2. V. V. Edara, C. Norwood, K. Floyd, L. Lai, M. E. Davis-Gardner, W. H. Hudson, G. Mantus, L. E. Nyhoff, M. W. Adelman, R. Fineman, S. Patel, R. Byram, D. N. Gomes, G. Michael, H. Abdullahi, N. Beydoun, B. Panganiban, N. McNair, K. Hellmeister, J. Pitts, J. Winters, J. Kleinhenz, J. Usher, J. B. O'Keefe, A. Piantadosi, J. J. Waggoner, A. Babiker, D. S. Stephens, E. J. Anderson, S. Edupuganti, N. Rouphael, R. Ahmed, J. Wrammert, M. S. Suthar, Infection- and vaccine-induced antibody binding and neutralization of the B.1.351 SARS-CoV-2 variant. *Cell Host Microbe* **29**, 516–521.E3 (2021). doi:10.1016/j.chom.2021.03.009 Medline

3. M. Hoffmann, P. Arora, R. Groß, A. Seidel, B. F. Hörnich, A. S. Hahn, N. Krüger, L. Graichen, H. Hofmann-Winkler, A. Kempf, M. S. Winkler, S. Schulz, H.-M. Jäck, B. Jahrsdörfer, H. Schrezenmeier, M. Müller, A. Kleger, J. Münch, S. Pöhlmann, SARS-CoV-2 variants B.1.351 and P.1 escape from neutralizing antibodies. *Cell* **184**, 2384–2393.E12 (2021). doi:10.1016/j.cell.2021.03.036 Medline

4. X. Shen, H. Tang, R. Pajon, G. Smith, G. M. Glenn, W. Shi, B. Korber, D. C. Montefiori, Neutralization of SARS-CoV-2 Variants B.1.429 and B.1.351. *N. Engl. J. Med.* **384**, 2352–2354 (2021). doi:10.1056/NEJMc2103740 Medline

5. P. Wang, M. S. Nair, L. Liu, S. Iketani, Y. Luo, Y. Guo, M. Wang, J. Yu, B. Zhang, P. D. Kwong, B. S. Graham, J. R. Mascola, J. Y. Chang, M. T. Yin, M. Sobieszczyk, C. A. Kyratsous, L. Shapiro, Z. Sheng, Y. Huang, D. D. Ho, Antibody resistance of SARS-CoV-2 variants B.1.351 and B.1.1.7. *Nature* **593**, 130–135 (2021). doi:10.1038/s41586-021-03398-2 Medline

6. C. K. Wibmer, F. Ayres, T. Hermanus, M. Madzivhandila, P. Kgagudi, B. Oosthuysen, B. E. Lambson, T. de Oliveira, M. Vermeulen, K. van der Berg, T. Rossouw, M. Boswell, V. Ueckermann, S. Meiring, A. von Gottberg, C. Cohen, L. Morris, J. N. Bhiman, P. L. Moore, SARS-CoV-2 501Y.V2 escapes neutralization by South African COVID-19 donor plasma. *Nat. Med.* **27**, 622–625 (2021). doi:10.1038/s41591-021-01285-x Medline

7. K. Wu, A. P. Werner, M. Koch, A. Choi, E. Narayanan, G. B. E. Stewart-Jones, T. Colpitts, H. Bennett, S. Boyoglu-Barnum, W. Shi, J. I. Moliva, N. J. Sullivan, B. S. Graham, A. Carfi, K. S. Corbett, R. A. Seder, D. K. Edwards, Serum Neutralizing Activity Elicited by mRNA-1273 Vaccine. *N. Engl. J. Med.* **384**, 1468–1470 (2021). doi:10.1056/NEJMc2102179 Medline

8. D. H. Barouch, K. E. Stephenson, J. Sadoff, J. Yu, A. Chang, M. Gebre, K. McMahan, J. Liu, A. Chandrashekar, S. Patel, M. Le Gars, A. M. de Groot, D. Heerwegh, F. Struyf, M. Douoguih, J. van Hoof, H. Schuitemaker, Durable Humoral and Cellular Immune Responses 8 Months after Ad26.COV2.S Vaccination. *N. Engl. J. Med.* 10.1056/NEJMc2108829 (2021). doi:10.1056/NEJMc2108829 Medline

9. N. Zhu, D. Zhang, W. Wang, X. Li, B. Yang, J. Song, X. Zhao, B. Huang, W. Shi, R. Lu, P. Niu, F. Zhan, X. Ma, D. Wang, W. Xu, G. Wu, G. F. Gao, W. Tan, China Novel Coronavirus Investigating and Research Team, A Novel Coronavirus from Patients with

Pneumonia in China, 2019. *N. Engl. J. Med.* **382**, 727–733 (2020). doi:10.1056/NEJMoa2001017 Medline

10. L. J. Abu-Raddad, H. Chemaitelly, A. A. Butt, National Study Group for COVID-19 Vaccination, Effectiveness of the BNT162b2 Covid-19 Vaccine against the B.1.1.7 and B.1.351 Variants. *N. Engl. J. Med.* **385**, 187–189 (2021). doi:10.1056/NEJMc2104974 Medline

11. N. Dagan, N. Barda, E. Kepten, O. Miron, S. Perchik, M. A. Katz, M. A. Hernán, M. Lipsitch, B. Reis, R. D. Balicer, BNT162b2 mRNA Covid-19 Vaccine in a Nationwide Mass Vaccination Setting. *N. Engl. J. Med.* **384**, 1412–1423 (2021). doi:10.1056/NEJMoa2101765 Medline

12. A. Sheikh, J. McMenamin, B. Taylor, C. Robertson, Public Health Scotland and the EAVE II Collaborators, SARS-CoV-2 Delta VOC in Scotland: Demographics, risk of hospital admission, and vaccine effectiveness. *Lancet* **397**, 2461–2462 (2021). doi:10.1016/S0140-6736(21)01358-1 Medline

13. N. Doria-Rose, M. S. Suthar, M. Makowski, S. O'Connell, A. B. McDermott, B. Flach, J. E. Ledgerwood, J. R. Mascola, B. S. Graham, B. C. Lin, S. O'Dell, S. D. Schmidt, A. T. Widge, V.-V. Edara, E. J. Anderson, L. Lai, K. Floyd, N. G. Rouphael, V. Zarnitsyna, P. C. Roberts, M. Makhene, W. Buchanan, C. J. Luke, J. H. Beigel, L. A. Jackson, K. M. Neuzil, H. Bennett, B. Leav, J. Albert, P. Kunwar, mRNA-1273 Study Group, Antibody Persistence through 6 Months after the Second Dose of mRNA-1273 Vaccine for Covid-19. *N. Engl. J. Med.* **384**, 2259–2261 (2021). doi:10.1056/NEJMc2103916 Medline

14. A. T. Widge, N. G. Rouphael, L. A. Jackson, E. J. Anderson, P. C. Roberts, M. Makhene, J. D. Chappell, M. R. Denison, L. J. Stevens, A. J. Pruijssers, A. B. McDermott, B. Flach, B. C. Lin, N. A. Doria-Rose, S. O'Dell, S. D. Schmidt, K. M. Neuzil, H. Bennett, B. Leav, M. Makowski, J. Albert, K. Cross, V.-V. Edara, K. Floyd, M. S. Suthar, W. Buchanan, C. J. Luke, J. E. Ledgerwood, J. R. Mascola, B. S. Graham, J. H. Beigel, mRNA-1273 Study Group, Durability of Responses after SARS-CoV-2 mRNA-1273 Vaccination. *N. Engl. J. Med.* **384**, 80–82 (2021). doi:10.1056/NEJMc2032195 Medline

15. E. J. Anderson, N. G. Rouphael, A. T. Widge, L. A. Jackson, P. C. Roberts, M. Makhene, J. D. Chappell, M. R. Denison, L. J. Stevens, A. J. Pruijssers, A. B. McDermott, B. Flach, B. C. Lin, N. A. Doria-Rose, S. O'Dell, S. D. Schmidt, K. S. Corbett, P. A. Swanson 2nd, M. Padilla, K. M. Neuzil, H. Bennett, B. Leav, M. Makowski, J. Albert, K. Cross, V. V. Edara, K. Floyd, M. S. Suthar, D. R. Martinez, R. Baric, W. Buchanan, C. J. Luke, V. K. Phadke, C. A. Rostad, J. E. Ledgerwood, B. S. Graham, J. H. Beigel, mRNA-1273 Study Group, Safety and Immunogenicity of SARS-CoV-2 mRNA-1273 Vaccine in Older Adults. *N. Engl. J. Med.* **383**, 2427–2438 (2020). doi:10.1056/NEJMoa2028436 Medline

16. L. A. Jackson, E. J. Anderson, N. G. Rouphael, P. C. Roberts, M. Makhene, R. N. Coler, M. P. McCullough, J. D. Chappell, M. R. Denison, L. J. Stevens, A. J. Pruijssers, A. McDermott, B. Flach, N. A. Doria-Rose, K. S. Corbett, K. M. Morabito, S. O'Dell, S. D. Schmidt, P. A. Swanson 2nd, M. Padilla, J. R. Mascola, K. M. Neuzil, H. Bennett, W. Sun, E. Peters, M. Makowski, J. Albert, K. Cross, W. Buchanan, R. Pikaart-Tautges, J. E. Ledgerwood, B. S. Graham, J. H. Beigel, mRNA-1273 Study Group, An mRNA Vaccine against SARS-CoV-2 - Preliminary Report. *N. Engl. J. Med.* **383**, 1920–1931 (2020). doi:10.1056/NEJMoa2022483 Medline

17. A. Vanderheiden, V. V. Edara, K. Floyd, R. C. Kauffman, G. Mantus, E. Anderson, N. Rouphael, S. Edupuganti, P.-Y. Shi, V. D. Menachery, J. Wrammert, M. S. Suthar, Development of a Rapid Focus Reduction Neutralization Test Assay for Measuring SARS-CoV-2 Neutralizing Antibodies. *Curr. Protoc. Immunol.* **131**, e116 (2020). doi:10.1002/cpim.116 Medline

18. D. Weissman, M.-G. Alameh, T. de Silva, P. Collini, H. Hornsby, R. Brown, C. C. LaBranche, R. J. Edwards, L. Sutherland, S. Santra, K. Mansouri, S. Gobeil, C. McDanal, N. Pardi, N. Hengartner, P. J.C. Lin, Y. Tam, P. A. Shaw, M. G. Lewis, C. Boesler, U. Şahin, P. Acharya, B. F. Haynes, B. Korber, D. C. Montefiori, D614G Spike Mutation Increases SARS CoV-2 Susceptibility to Neutralization. *Cell Host Microbe* **29**, 23–31.E4 (2021). doi:10.1016/j.chom.2020.11.012 Medline

19. V. V. Edara, W. H. Hudson, X. Xie, R. Ahmed, M. S. Suthar, Neutralizing Antibodies Against SARS-CoV-2 Variants After Infection and Vaccination. *JAMA* **325**, 1896–1898 (2021). doi:10.1001/jama.2021.4388 Medline

20. L. Wang, T. Zhou, Y. Zhang, E. S. Yang, C. A. Schramm, W. Shi, A. Pegu, O. K. Oloniniyi, A. R. Henry, S. Darko, S. R. Narpala, C. Hatcher, D. R. Martinez, Y. Tsybovsky, E. Phung, O. M. Abiona, A. Antia, E. M. Cale, L. A. Chang, M. Choe, K. S. Corbett, R. L. Davis, A. T. DiPiazza, I. J. Gordon, S. Helmold Hait, T. Hermanus, P. Kgagudi, F. Laboune, K. Leung, T. Liu, R. D. Mason, A. F. Nazzari, L. Novik, S. O'Connell, S. O'Dell, A. S. Olia, S. D. Schmidt, T. Stephens, C. D. Stringham, C. A. Talana, I.-T. Teng, D. A. Wagner, A. T. Widge, B. Zhang, M. Roederer, J. E. Ledgerwood, T. J. Ruckwardt, M. R. Gaudinski, P. L. Moore, N. A. Doria-Rose, R. S. Baric, B. S. Graham, A. B. McDermott, D. C. Douek, P. D. Kwong, J. R. Mascola, N. J. Sullivan, J. Misasi, Ultrapotent antibodies against diverse and highly transmissible SARS-CoV-2 variants. *Science* **373**, eabh1766 (2021). doi:10.1126/science.abh1766 Medline

21. D. Wrapp, N. Wang, K. S. Corbett, J. A. Goldsmith, C.-L. Hsieh, O. Abiona, B. S. Graham, J. S. McLellan, Cryo-EM structure of the 2019-nCoV spike in the prefusion conformation. *Science* **367**, 1260–1263 (2020). doi:10.1126/science.abb2507 Medline

22. L. R. Baden, H. M. El Sahly, B. Essink, K. Kotloff, S. Frey, R. Novak, D. Diemert, S. A. Spector, N. Rouphael, C. B. Creech, J. McGettigan, S. Khetan, N. Segall, J. Solis, A. Brosz, C. Fierro, H. Schwartz, K. Neuzil, L. Corey, P. Gilbert, H. Janes, D. Follmann, M. Marovich, J. Mascola, L. Polakowski, J. Ledgerwood, B. S. Graham, H. Bennett, R. Pajon, C. Knightly, B. Leav, W. Deng, H. Zhou, S. Han, M. Ivarsson, J. Miller, T. Zaks, COVE Study Group, Efficacy and Safety of the mRNA-1273 SARS-CoV-2 Vaccine. *N. Engl. J. Med.* **384**, 403–416 (2021). doi:10.1056/NEJMoa2035389 Medline

23. R. Bayarri-Olmos, A. Rosbjerg, L. B. Johnsen, C. Helgstrand, T. Bak-Thomsen, P. Garred, M.-O. Skjoedt, The SARS-CoV-2 Y453F mink variant displays a pronounced increase in ACE-2 affinity but does not challenge antibody neutralization. *J. Biol. Chem.* **296**, 100536 (2021). doi:10.1016/j.jbc.2021.100536 Medline

24. M. Hoffmann, L. Zhang, N. Krüger, L. Graichen, H. Kleine-Weber, H. Hofmann-Winkler, A. Kempf, S. Nessler, J. Riggert, M. S. Winkler, S. Schulz, H.-M. Jäck, S. Pöhlmann, SARS-CoV-2 mutations acquired in mink reduce antibody-mediated neutralization. *Cell Rep.* **35**, 109017 (2021). doi:10.1016/j.celrep.2021.109017 Medline

25. T. Tada, B. M. Dcosta, H. Zhou, A. Vaill, W. Kazmierski, N. R. Landau, Decreased neutralization of SARS-CoV-2 global variants by therapeutic anti-spike protein monoclonal antibodies. bioRxiv 2021.02.18.431897 [Preprint] (2021). https://doi.org/10.1101/2021.02.18.431897.

26. A. Ciabattini, C. Nardini, F. Santoro, P. Garagnani, C. Franceschi, D. Medaglini, Vaccination in the elderly: The challenge of immune changes with aging. *Semin. Immunol.* **40**, 83–94 (2018). doi:10.1016/j.smim.2018.10.010 Medline

27. Z. Wang, F. Muecksch, D. Schaefer-Babajew, S. Finkin, C. Viant, C. Gaebler, H.-H. Hoffmann, C. O. Barnes, M. Cipolla, V. Ramos, T. Y. Oliveira, A. Cho, F. Schmidt, J. Da Silva, E. Bednarski, L. Aguado, J. Yee, M. Daga, M. Turroja, K. G. Millard, M. Jankovic, A. Gazumyan, Z. Zhao, C. M. Rice, P. D. Bieniasz, M. Caskey, T. Hatziioannou, M. C. Nussenzweig, Naturally enhanced neutralizing breadth against SARS-CoV-2 one year after infection. *Nature* **595**, 426–431 (2021). doi:10.1038/s41586-021-03696-9 Medline

28. K. Wu, A. Choi, M. Koch, L. Ma, A. Hill, N. Nunna, W. Huang, J. Oestreicher, T. Colpitts, H. Bennett, H. Legault, Y. Paila, B. Nestorova, B. Ding, R. Pajon, J. M. Miller, B. Leav, A. Carfi, R. McPhee, D. K. Edwards, Preliminary Analysis of Safety and Immunogenicity of a SARS-CoV-2 Variant Vaccine Booster. medRxiv 2021.2005.2005.21256716 [Preprint] (2021). https://doi.org/10.1101/2021.05.05.21256716.

29. L. Naldini, U. Blömer, F. H. Gage, D. Trono, I. M. Verma, Efficient transfer, integration, and sustained long-term expression of the transgene in adult rat brains injected with a lentiviral vector. *Proc. Natl. Acad. Sci. U.S.A.* **93**, 11382–11388 (1996). doi:10.1073/pnas.93.21.11382 Medline

30. E. Böttcher, T. Matrosovich, M. Beyerle, H.-D. Klenk, W. Garten, M. Matrosovich, Proteolytic activation of influenza viruses by serine proteases TMPRSS2 and HAT from human airway epithelium. *J. Virol.* **80**, 9896–9898 (2006). doi:10.1128/JVI.01118-06 Medline

31. L. C. Katzelnick, A. Coello Escoto, B. D. McElvany, C. Chávez, H. Salje, W. Luo, I. Rodriguez-Barraquer, R. Jarman, A. P. Durbin, S. A. Diehl, D. J. Smith, S. S. Whitehead, D. A. T. Cummings, Viridot: An automated virus plaque (immunofocus) counter for the measurement of serological neutralizing responses with application to dengue virus. *PLOS Negl. Trop. Dis.* **12**, e0006862 (2018). doi:10.1371/journal.pntd.0006862 Medline

32. H. Liu, Q. Zhang, P. Wei, Z. Chen, K. Aviszus, J. Yang, W. Downing, C. Jiang, B. Liang, L. Reynoso, G. P. Downey, S. K. Frankel, J. Kappler, P. Marrack, G. Zhang, The basis of a more contagious 501Y.V1 variant of SARS-CoV-2. *Cell Res.* **31**, 720–722 (2021). doi:10.1038/s41422-021-00496-8 Medline
