# Supplementary material for: Durability of mRNA-1273 vaccine–induced antibodies against SARS-CoV-2 variants
Source: Science. 2021 Aug 12;373(6561):1372–7. doi: 10.1126/science.abj4176 (PMC8691522; doi:10.1126/science.abj4176)
Supplement: Supplementary file 3 — MDAR Reproducibility Checklist [file science.abj4176_mdar_reproducibility_checklist.pdf]

Materials Design Analysis Reporting (MDAR) Checklist for Authors

The MDAR framework establishes a minimum set of requirements in transparent reporting applicable to studies in the life sciences (see Statement of Task: doi:10.31222/osf.io/9sm4x.). The MDAR checklist is a tool for authors, editors and others seeking to adopt the MDAR framework for transparent reporting in manuscripts and other outputs. Please refer to the MDAR Elaboration Document for additional context for the MDAR framework.

Materials

**Antibodies**	**Yes (indicate where provided: page no/section/legend)**	**n/a**
For commercial reagents, provide supplier name, catalogue number and RRID, if available.	Yes, page 4 supplement	

**Cell materials**	**Yes (indicate where provided: page no/section/legend)**	**n/a**
**Cell lines:** Provide species information, strain. Provide accession number in repository **OR** supplier name, catalog number, clone number, **OR** RRID	Yes, page 2 supplement	
**Primary cultures:** Provide species, strain, sex of origin, genetic modification status.	n/a	

**Experimental animals**	**Yes (indicate where provided: page no/section/legend)**	**n/a**
**Laboratory animals:** Provide species, strain, sex, age, genetic modification status. Provide accession number in repository **OR** supplier name, catalog number, clone number, **OR** RRID	n/a	
**Animal observed in or captured from the field:** Provide species, sex and age where possible	n/a	
**Model organisms:** Provide Accession number in repository (where relevant) **OR** RRID	n/a	

**Plants and microbes**	**Yes (indicate where provided: page no/section/legend)**	**n/a**
**Plants:** provide species and strain, unique accession number if available, and source (including location for collected wild specimens)	n/a	
**Microbes:** provide species and strain, unique accession number if available, and source	n/a	

**Human research participants**	**Yes (indicate where provided: page no/section/legend)**	**n/a**
Identify authority granting ethics approval (IRB or equivalent committee(s), provide reference number for approval.	Yes, page 2 supplement	
Provide statement confirming informed consent obtained from study participants.	Yes, page 2 supplement	
Report on age and sex for all study participants.	No	

Design

Study protocol	Yes (indicate where provided: page no/section/legend)	n/a
For clinical trials, provide the trial registration number **OR** cite DOI in manuscript.	Yes, page 2 supplement	

Laboratory protocol	Yes (indicate where provided: page no/section/legend)	n/a
Provide DOI or other citation details if detailed step-by-step protocols are available.	Yes, all protocols detailed or referenced in supplement methods section	

Experimental study design (statistics details)	Yes (indicate where provided: page no/section/legend)	n/a
State whether and how the following have been done**, or** if they were not carried out.		
Sample size determination	As described in references 21-22	
Randomisation		
Blinding		
Inclusion/exclusion criteria		

Sample definition and in-laboratory replication	Yes (indicate where provided: page no/section/legend)	n/a
State number of times the experiment was replicated in laboratory	Yes, stated in supplement section for each method	
Define whether data describe technical or biological replicates	Yes, data are technical replicates	

Ethics	Yes (indicate where provided: page no/section/legend)	n/a
Studies involving human participants: State details of authority granting ethics approval (IRB or equivalent committee(s), provide reference number for approval.	Yes, page 2 supplement	
Studies involving experimental animals: State details of authority granting ethics approval (IRB or equivalent committee(s), provide reference number for approval.	n/a	
Studies involving specimen and field samples: State if relevant permits obtained, provide details of authority approving study; if none were required, explain why.	n/a	

Dual Use Research of Concern (DURC)	Yes (indicate where provided: page no/section/legend)	n/a
If study is subject to dual use research of concern, state the authority granting approval and reference number for the regulatory approval	n/a	

Analysis

Attrition	Yes (indicate where provided: page no/section/legend)	n/a
State if sample or data point from the analysis is excluded, and whether the criteria for exclusion were determined and specified in advance.	n/a	

Statistics	Yes (indicate where provided: page no/section/legend)	n/a
Describe statistical tests used and justify choice of tests.	Yes supplement section page 4.	

Data Availability	Yes (indicate where provided: page no/section/legend)	n/a
State whether newly created datasets are available, including protocols for access or restriction on access.	n/a	
If data are publicly available, provide accession number in repository or DOI or URL.	n/a	
If publicly available data are reused, provide accession number in repository or DOI or URL, where possible.	n/a	

Code Availability	Yes (indicate where provided: page no/section/legend)	n/a
For all newly generated code and software essential for replicating the main findings of the study:		
State whether the code or software is available.	n/a	
If code is publicly available, provide accession number in repository, or DOI or URL.	n/a	

Reporting

Adherence to community standards	Yes (indicate where provided: page no/section/legend)	n/a
MDAR framework recommends adoption of discipline-specific guidelines, established and endorsed through community initiatives. Journals have their own policy about requiring specific guidelines and recommendations to complement MDAR.		
State if relevant guidelines (eg., ICMJE, MIBBI, ARRIVE) have been followed, and whether a checklist (eg., CONSORT, PRISMA, ARRIVE) is provided with the manuscript.	n/a	
